# Residues from black soldier fly (*Hermetia illucens*) larvae rearing influence the plant-associated soil microbiome in the short term

**DOI:** 10.3389/fmicb.2022.994091

**Published:** 2022-09-26

**Authors:** Adrian Fuhrmann, Benjamin Wilde, Rafaela Feola Conz, Speciose Kantengwa, Matieyedou Konlambigue, Barthazar Masengesho, Kokou Kintche, Kinfe Kassa, William Musazura, Leonhard Späth, Moritz Gold, Alexander Mathys, Johan Six, Martin Hartmann

**Affiliations:** ^1^Sustainable Agroecosystems Group, Institute of Agricultural Sciences, Department of Environmental Systems Science, ETH Zürich, Zürich, Switzerland; ^2^Singapore-ETH Centre, Singapore, Singapore; ^3^International Institute of Tropical Agriculture, Kigali, Rwanda; ^4^Maggot Farm Production Ltd., Kamonyi, Kamonyi, Rwanda; ^5^Faculty of Water Supply and Environmental Engineering, Arba Minch University, Arba Minch, Ethiopia; ^6^School of Agricultural, Earth and Environmental Sciences, University of Kwazulu-Natal, Pietermaritzburg, South Africa; ^7^Transdisciplinary Lab, Department of Environmental Systems Science, ETH Zürich, Zürich, Switzerland; ^8^Sustainable Food Processing Laboratory, Institute of Food, Nutrition and Health, Department of Health Science and Technology, ETH Zürich, Zürich, Switzerland,; ^9^Department of Sanitation, Water and Solid Waste for Development (Sandec), Swiss Federal Institute of Aquatic Science and Technology, Dübendorf, Switzerland

**Keywords:** *Hermentia illucens*, soil microbiome, organic fertilizers, frass, plant growth promotion, circular economy, black soldier fly larvae

## Abstract

The larvae of the black soldier fly (BSFL, *Hermetia illucens*) efficiently close resource cycles. Next to the nutrient-rich insect biomass used as animal feed, the residues from the process are promising plant fertilizers. Besides a high nutrient content, the residues contain a diverse microbial community and application to soil can potentially promote soil fertility and agricultural production through the introduction of beneficial microbes. This research assessed the application of the residues on plant-associated bacterial and fungal communities in the rhizosphere of a grass-clover mix in a 42-day greenhouse pot study. Potted soil was amended with BSFL residues (BR+) or conventional compost (CC+) produced by Rwandan waste management companies in parallel to residues and compost sterilized (BR-, CC-) by high-energy electron beam (HEEB) as abiotic controls. The fertilizers were applied at a rate of 150  kg N  ha^−1^. Soil bacterial and fungal communities in both fertilizer and soil were assessed by high-throughput sequencing of ribosomal markers at different times after fertilizer application. Additionally, indicators for soil fertility such as basal respiration, plant yield and soil physicochemical properties were analyzed. Results showed that the application of BSFL residues influenced the soil microbial communities, and especially fungi, stronger than CC fertilizers. These effects on the microbial community structure could partly be attributed to a potential introduction of microbes to the soil by BSFL residues (e.g., members of genus *Bacillus*) since untreated and sterilized BSFL residues promoted different microbial communities. With respect to the abiotic effects, we emphasize a potential driving role of particular classes of organic matter like fiber and chitin. Indeed, especially taxa associated with decomposition of organic matter (e.g., members of the fungal genus *Mortierella*) were promoted by the application of BSFL residues. Soil fertility with respect to plant yield (+17% increase compared to unamended control) and basal respiration (+16% increase compared to unamended control) tended to be improved with the addition of BSFL residues. Findings underline the versatile opportunities for soil fertility arising from the application of BSFL residues in plant production and point to further research on quantification of the described effects.

## Introduction

The intensification of ecological imbalances combined with a growing world population poses an enormous challenge for the global society of the 21st century. The demand for food is steadily increasing, and sustainable approaches are needed to solve the resulting challenges in agricultural production (McKenzie and Williams, 2015). The soil microbiome is essential to the viability of global ecosystems, since it is a major driver of soil structure formation, organic matter breakdown, nutrient provisioning, plant growth promotion, and stress and disease control (Barrios, 2007). Its functional capacity to influence soil fertility and thus crop performance (Van Der Heijden et al., 2016; Saleem et al., 2019) has gained increasing attention in sustainable food production. Intentionally steering the soil microbiome by adapting agricultural management practices is considered a promising approach to increasing the resilience of a global agriculture facing environmental challenges (Wang and Haney, 2020). For example, Hartmann et al. (2015) showed that the type of agricultural practice and especially the type of applied fertilizer can play a crucial role in shaping long-term soil bacterial and fungal communities in agroecosystems. Sun et al. (2016) further indicated that exogenous microorganisms associated with organic fertilizers such as animal manure can establish and alter the soil microbiome. Compost fertilizer is a promising source of exogenous microorganisms with nutrient-cycling and disease-suppressive capacities that can improve soil health (Lutz et al., 2020). Although organic sources of fertilizer such as compost have demonstrated positive impacts on the soil microbiome, providing sufficient quantities of compost is a major impediment to sustainable agricultural production.

The utilization of insects in the agri-food-chain is seen as a promising way to generate valuable protein feed and fertilizer in a more sustainable way. Van Huis and Oonincx (2017) pointed out that insects emit less greenhouse gases, require less land, convert inputs more efficiently than conventional livestock, and can feed on organic side-streams. Besides rearing insects directly for human consumption, invertebrates are suitable to become a replacement for products associated with comparably negative environmental externalities like soy or fish based protein feed (Smetana et al., 2019). Especially the larva of the black soldier fly (BSFL, *Hermetia illucens*) is considered to have great potential for circular economy (Varelas, 2019), being able to metabolize a wide range of organic substances including human excreta (Banks et al., 2014) and to reduce dry weight of waste material by 27–72% (Gold et al., 2018). Since BSFL reproduce more efficiently in tropical regions, the technology offers an opportunity for innovation, particularly in low-income countries (Barragan-Fonseca et al., 2017). In addition to the sustainability potential of the larva, the extensively accruing residues from insect rearing, i.e., excreta, undigested substrates and shed exoskeletons, bear the chance of returning versatile services to the agri-food chain *via* their provision of nutrients, bioactive molecules, and potentially beneficial microbes when amended to the soil–plant system (Poveda, 2021; Barragán-Fonseca et al., 2022).

The fertilizer effect of BSFL residues (in some studies also referred to as “frass”) on plants has been subjected to several studies. Outcomes from greenhouse and field trials were mostly comparable or superior to mineral fertilization (Kebli and Sinaj, 2017; Klammsteiner et al., 2020a; Beesigamukama et al., 2020b; Anyega et al., 2021) with exceptions (Gärttling et al., 2020; Gebremikael et al., 2022). Chirere et al. (2021) pointed out that the generally higher nutrient contents of BSFL residues compared to other organic soil amendments make the residues an agronomically practical alternative. It should be noted that, depending on the substrates fed to BSFL, physicochemical properties of the residues may strongly differ (Schmitt and de Vries, 2020; Klammsteiner et al., 2020a), which results in distinct effects on soil fertility (Gebremikael et al., 2022). Furthermore, postprocessing of BSFL residues, especially composting, with potential influence on the composition of the residues, is not conducted consistently across studies. This variability in post-processing potentially also influences attributed toxic effects on the soil–plant system (Zorrilla and Hussain, 2020), whereas high concentrations of phenols in uncomposted residues were suggested to be one reason for a reduced germination rate of Pak Choi plants (Song et al., 2021). The question of composting insect residues for agricultural use is crucial for the insect industry because the requisite infrastructure bears significant economic and logistic implications. In fact, BSFL residues can reduce plant growth when applied in high amounts (≥ 15–20 t ha^−1^), whereas the generally high ammonium and salt content has been discussed as a potential reason (Temple et al., 2013; Alattar et al., 2016; Chiam et al., 2021). Chitin and its derivates stemming from molting BSFL are associated with plant performance promotion through inhibition of pathogenic and support of beneficial soil microbes as well as through a direct stimulation of plant growth (Sharp, 2013). The BSFL residues are likely to contain further bioactive compounds of importance for the soil microbiome, since BSFL genes encoding for several antimicrobial peptides and their diet-dependent expression were identified (Elhag et al., 2017; Vogel et al., 2018).

Poveda (2021) emphasized the structural and functional similarities between plant root and insect gut, suggesting not just an overlap of microbial communities between both habitats but also possible synergistic benefits when combined. As demonstrated in former studies, BSFL can substantially influence microbial community composition in its residues presumably *via* fecal transmission, leaving behind a characteristically imprinted residue (Gold et al., 2020b). When the organic amendment is introduced to the rhizosphere soil, the insect gut derived flora may merge with its plant-related analogue. For example, the microbiome of mealworm (*Tenebrio molitor*) excreta was reported to contain plant growth promoting bacterial taxa and to have a positive influence on the performance of chard plants when applied as fertilizer under greenhouse conditions (Poveda et al., 2019). Findings from Chiam et al. (2021) suggested that BSFL residues applied at high rates to soil (≥ 10% v/v BSFL residue) can change the soil bacterial community structure. Gebremikael et al. (2020) found in their soil incubation experiment that fertilization with BSFL residues produced from food waste (110 kg N ha^−1^) changed the microbial community significantly after 42 days based on an assessment with bacterial and fungal biomarkers. The authors suggested both microbes and chitin inherent to BSFL residues to be major drivers of soil microbial diversity.

Recently, Barragán-Fonseca et al. (2022) urged the clarification of the impact of insect residues on plant growth promoting rhizobacteria, crop growth and of potential underlying mechanisms. Several authors studying the application of insect residues in agricultural contexts suggest an indirect effect on the crop through an impact on the soil microbiome (Setti et al., 2019; Schmitt and de Vries, 2020; Beesigamukama et al., 2020a; Torgerson et al., 2021; Gebremikael et al., 2022; Tanga et al., 2022). The present pot study addressed the depicted knowledge gap. We sequenced the bacterial, archaeal and fungal community of the BSFL residues, and the rhizosphere fertilized with BSFL residues. The aim was to investigate the potential of the novel organic fertilizer to impact the plant-associated soil microbiome and mechanisms behind observed changes. By sterilizing soil amendments using high-energy electron beam (HEEB) radiation prior to their application, we sought to delineate the impact of biotic and abiotic components. We examined a possible transfer of species stemming from BSFL residues to the soil. To shed light on the consequences for soil fertility, we assessed changes in the soil microbial community and its responding microbial taxa as well as changes in plant yield and soil respiration. As a reference, we compared BSFL residues with a conventional compost as an established organic fertilizer.

## Materials and methods

### Organic fertilizers

The influence of the BSFL residues (BR) on the soil microbiome was assessed in a pot experiment by comparing a sterile and a non-sterile form (BR-, BR+) to conventional compost (CC) in a sterile and a non-sterile form (CC-, CC+). BR was provided by Maggot Farms Ltd. (Kamonyi, Rwanda), specialized in the bioconversion of organic waste-streams into animal feed with BSFL. Five day old larvae were added to a brewery waste-stream (spent malted barley grain from SKOL Brewery Ltd., Kigali, Rwanda). After around 14 days of feeding, first prepupae appeared and insects were separated from the residues. During the feeding process the waste material was reduced by 50–60% on a wet weight basis. BR was stored for 12 days in closed plastic bags at ambient temperature before shipping to Switzerland. CC was provided by COPED Ltd., a waste management company based in Kigali, Rwanda. The compost was produced with municipal waste (mainly organics: food waste, green waste; inorganic material that was not sorted out) and turned once during the composting time of around 6 months. The companies that provided the fertilizers are taking part in the RUNRES^1^ project which aims to improve circular food systems in city regions across Africa.

After sampling in Rwanda, fertilizers were stored in resealable polyethylene bags and either transported on ice or stored in the fridge at 5°C. After shipping to Switzerland, a fraction of both fertilizers was irradiated by HEEB radiation (Leoni Studer AG, Däniken, Switzerland) with a 10 MeV electron beam (Rhodotron TT300, IBA Corp., Louvain-la-Neuve, Belgium) at a dose of >32 kGy in accordance with the ISO 11137-3:2017 standard (International Organization for Standardization, 2017) as described before by Gold et al. (2020a). Sterilization was confirmed by conventional plate counting on tryptic soy agar (Sigma-Aldrich, Burlington, United States) at 25°C for 36 h. In technical triplicates, 2.5 g of each fertilizer was added to 22.5 ml of Milli-Q-water, vortexed, and 1 ml suspension was spread on an 8.8 cm diameter agar plate. Prior to application to the pots, fertilizers were gently crushed and homogenized by hand without opening the sealed polyethylene bags.

### Experimental soil

The experimental soil was collected at a non-managed grassland in Winterthur, Switzerland. The soil had a silt loam texture (Soil Science Division Staff, 2017) containing 53 g clay, 524 g silt and 424 g sand kg^−1^ soil. The neutral-to-slightly alkaline soil (pH 7.1) had an electrical conductivity (EC) of 450 μS cm^−1^ (see Table 1 for further characteristics). After collection, the soil was passed through a 4 mm sieve and stored at ambient temperature (≈15°C).

**Table 1:** Characteristics of the experimental soil.

Parameter ^1^	Content ^2^
EC [μS cm^−1^]	450
pH	7.1
N [g kg^−1^]	2.8 ± 0.1
C [g kg^−1^]	45.4 ± 1.7
C:N ratio	16.3 ± 1.0
P [g kg^−1^]	0.6 ± 0.0
K [g kg^−1^]	15.4 ± 0.1

1 EC (electrical conductivity), pH (in H_2_O), N (total nitrogen), C (total carbon), C:N ratio (ratio between C and N), P (total phosphorus), K (total potassium).

2 Values are based on dry weight (average ± standard deviation if *n* = 3).

### Pot experiment

The six-week long pot experiment was conducted in a climate-controlled greenhouse at the ETH Zurich research station (Lindau, Switzerland, 47°44′92.5”N 8°68′20.7″E) from January 8^th^ to February 19^th^, 2021. BR+, BR-, CC+, and CC- were well mixed with the experimental soil and placed into plastic pots (27 cm diameter x 22.5 cm height) with five repetitions per soil-fertilizer mixture (sBR+, sBR-, sCC+ and sCC-). Similarly, a no-fertilizer treatment (sN0) was prepared as a control without any amendment, amounting to a total of 25 experimental units (pots). The four different fertilizers (BR+, BR-, CR+, CR-) were applied to the experimental soil at a rate of 75 mg nitrogen (N) kg^−1^ dry weight (DW) of soil. That corresponds to a rate 150 kg N ha^−1^ given a bulk density of 1 g cm^−3^ and a 20 cm mixing zone of the upper soil layer in the field, following the approach of Klammsteiner et al. (2020a). Thus 15.9 g (13.9 g DW) BR, or 39.6 g (25.1 g DW) CC were thoroughly mixed with 7.5 kg (5.9 kg DW) of soil for each experimental unit separately and filled into the pots. The unsealed pots were lined at the bottom with synthetic felt inside to avoid loss of substrate. All pots were arranged in the greenhouse in a randomized complete block design containing five blocks with a size of 1 m × 1 m each (Supplementary Figure 1).

After a three-day equilibration phase, red clover (*Trifolium pratense* var. *Taifun*) and Italian ryegrass (*Loilium multiflorum var. Oryx*) seeds were sown at a ratio of 0.75 (g seed g^−1^ seed) respectively as suggested for two-year-lasting grass-clover-mixtures (Lehmann et al., 2000). The total seeding rate corresponded to 52.5 kg seeds ha^−1^. Temperatures in the greenhouse ranged from 14°C to 26°C, with an average of 17°C. Nine days after sowing, artificial light (400-W metal halide lamps, Hugentobler Spezialleuchten AG, Weinfelden, Switzerland) with a photoperiod of 14/10 h was imposed in addition to the natural light source in the greenhouse. Throughout the experiment, pots were watered three times a week with the same amount of purified water (reverse osmosis) per pot. Watering targeted a water filled pore space of 60% (Haney and Haney, 2010). Weeds were removed manually on a weekly basis.

Soil samples were collected on day 0, day 24 and day 42 following equilibration. Soil samples were collected over the whole soil profile (0–17 cm) with a steel core soil sampler (diameter of 1.3 cm), collecting at least three soil cores from each pot. Coring locations were evenly distributed over the pot surface. Soil samples were immediately stored in resealable polyethylene bags and stored on ice until further processing and manual removal of plant roots. Sampling cores were re-filled with autoclaved quartz sand (0.4–1.2 mm). After 42 days, plant shoot biomass was harvested for each pot, weighed, dried at 60°C for 96 h, and weighed again to calculate wet and dry weight yield (DWY) respectively. After 42 days, roots thoroughly permeated the entire pot such that all soil can be considered as rhizosphere (i.e., directly influenced by the roots). Throughout the experiment, none of the plants showed symptoms of nutrient deficiency, drought stress, or disease.

### Physicochemical analysis of fertilizers and soil

Fertilizers and soil samples were analyzed for water and DW content, EC, pH, nitrate (NO_3_^−^), and ammonium (NH_4_^+^). Elemental composition was determined for fertilizers and untreated experimental soil only. To assess gravimetric water content (GWC) and DW, 5 g of fertilizer or soil were dried at 105°C for 72 h. EC and pH in soil samples were determined in a suspension of 10 g of dried soil (40°C, 72 h) and Milli-Q-water (1:2.5) using a multi-parameter meter (pHenomonal^®^ MU6100L, VWR, Radnor, United States) after shaking for 24 h. EC and pH in fertilizer samples was determined in a suspension with a ratio of 1:5 in Milli-Q-water, because of strong water absorption, and shaken for just 1 hour to avoid fermentation.

Inorganic N was determined colorimetrically (NO_3_^−^: 540 nm, NH_4_^+^: 650 nm, V-1200, VWR) following (Forster, 1995) and Doane and Horwáth (2003) after extraction by the addition of 50 ml KCl (2 M) to 10 g of wet fertilizer or soil sample, 1 h of shaking and filtration through ashless filters (Grade 42, Whatman, Little Chalfont). Total mineral N was estimated as the sum of NO_3_^−^-N and NH_4_^+^-N. Total C and N were determined using 200 mg dried (40°C, 72 h) and milled (MM200, Retsch GmbH, Haan, Germany) sample using a C/N analyzer (CN628, LECO Corporation, St. Joseph, United States).

Phosphorus (P), potassium (K), calcium (Ca), magnesium (Mg), sodium (Na), iron (Fe), zinc (Zn), manganese (Mn), aluminum (Al), copper (Cu) and cadmium (Cd) were determined with ICP-OES (5,100 SVDV, Agilent Technologies, Santa Clara, United States) after digestion of 200 mg dried (40°C, 72 h) and milled (MM200, Retsch GmbH) fertilizer or 1,000 mg of dried (40°C, 72 h) and milled (MM200, Retsch GmbH) experimental soil sample. Fertilizer samples were digested with 15 ml HNO_3_ (65%) for 90 min at 120°C, and 3 ml H_2_O_2_ (30%) of 90 min at 120°C (DigiPREP MS, Baie-D’Urfé, Canada). The experimental soil was mixed with 2 ml of Milli-Q-water and digested with 2 ml HNO_3_ (70%) and 6 ml HCl (37%) at 120°C for 90 min (DigiPREP MS, Baie-D’Urfé, Canada). Digests were filtered through ashless filters (Grade 41, Whatman) before ICP-OES analysis. For quantification of effective cation exchange capacity (CEC_eff_) and base saturation, based on Hendershot and Duquette (1986), 25 ml of BaCl_2_ (0.1 M) were added to 3 g of dried (40°C, 72 h) and milled (MM200, Retsch GmbH) soil and shaken for 2 h. After filtering through ashless filters (Grade 41, Whatman) exchangeable cations (Ca, Mg, Na, K, Al, Mn, and Fe) were analyzed with ICP-OES (5,100 SVDV, Agilent Technologies).

### Basal soil respiration

At the end of the pot experiment (day 42) soil samples were analyzed for basal respiration. 30 g of soil were incubated at room temperature (≈22°C, 12 h) in valved, air-proof jars (7.4 cm diameter × 10 cm height). Concentrations of CO_2_ emitted to the headspace of jars *via* soil basal respiration were determined using a LI-840A infrared gas analyzer (Li-Cor Inc., Lincoln, United States). It was integrated into a continuous flow system built according to Carbone et al. (2019) including the Flux Puppy app (v1.0.0). After flushing with ambient air, jars were successively connected to the system for each sampling (logging period: 60 s, logging interval: 1 s) within four consecutive sampling series (series interval: 4 h). Calculation of CO_2_ production by soil microbial respiration was performed based on Curiel Yuste et al. (2007). In brief, first means of CO_2_ concentrations (ppm) over the logging period were calculated for each timepoint. These concentrations were corrected for temperature changes over the duration of the experiment. Further, a correction for an inevitable dilution with ambient air present in the system (tubes, internal optical cell) upon each measurement was conducted. CO_2_ efflux (μmol g^−1^ h^−1^) was estimated from slopes of regression models applied to the generated CO_2_ concentration time series on each experimental unit taking into account jar volume and DW content of soil.

### Microbial community structure in fertilizers and soil

Bacterial, archaeal, and fungal community structure in fertilizers and soil was determined using a metabarcoding approach targeting ribosomal markers. For this purpose, samples were kept on ice for direct processing or stored frozen at – 20°C. All samples were exposed to not more than one freezing and thawing cycle before the DNA extraction from 250 mg of wet soil or fertilizer (DNeasy^®^ PowerSoil^®^ Pro Kit, QIAGEN, Hilden, Germany). Processing followed the manufacturer’s instructions and included a bead beating step (FastPrep-24^™^ 5G, MP Biomedical, Santa Ana, United States) running for two cycles of 60 s in total. Additional PCR-grade-water (VWR) was added to the three samples drawn from each fertilizer before bead beating, because of strong absorption of the lysis buffer from the DNeasy^®^ PowerSoil^®^ Pro Kit. Purity and quantity of extracted nucleic acids were analyzed by Uv–Vis spectrophotometry (Qiaxpert, QIAGEN). DNA concentration of each sample was normalized to 10 ng μL^−1^ using a QIAgility system (QIAGEN).

PCR amplification of the bacterial and archaeal (V3-V4 region of the 16S rRNA gene) and fungal (ITS2 region of the rrn operon) markers were conducted using primers 341F (5′–CCTAYGGGDBGCWSCAG-3′) and 806R (5′-GGACTACNVGGGTHTCTAAT-3′) for the bacterial/archaeal marker (Frey et al., 2016) and ITS3ngs (5′-CANCGATGAAGAACGYRG-3′) and ITS4ngs (5′-CCTSCSCTTANTDATATGC-3′) for the fungal markers (Tedersoo and Lindahl, 2016), respectively. PCR amplification was performed in a mixture containing 40 ng of template DNA, 0.4 μM of each primer (Microsynth, Balgach, Switzerland), 1x GoTaq^®^ Colorless Master Mix (Promega, Madison, WI, United States), and 0.5 mM MgCl_2_ (Promega) in a total reaction volume of 25 μl. Thermocycling consisted of an initial denaturation step (95°C, 2 min) followed by 30 (bacteria/archaea) or 35 (fungi) cycles of denaturation (95°C, 40 s), annealing (58°C for bacteria/archaea and 55°C for fungi, 40 s), and elongation (72°C for 1 min), and a final elongation step at 72°C for 10 min (C1000 Touch Thermal Cycler, BioRad Laboratories, Hercules, United States). Markers were amplified in three technical replicates, checked with gel electrophoreses (QIAxcel Advanced, Qiagen), and pooled prior to sequencing. PCR products were sent to the Functional Genomics Center Zurich (FGCZ, Zurich, Switzerland) for indexing PCR. Indexed PCR products were purified, quantified, and pooled in equal concentrations before preliminary sequencing on the Illumina MiniSeq platform (Illumina Inc., San Diego, United States) to inform library re-pooling for optimal equimolarity across samples. Final sequencing was conducted using the v3 chemistry (PE300) on the Illumina MiSeq platform (Illumina Inc.).

### Bioinformatics

Data obtained from sequencing were processed using a customized pipeline based on VSEARCH v.2.17.0 (Rognes et al., 2016) as described previously (Longepierre et al., 2021, 2022). Briefly, PhiX contaminants were removed by aligning reads to the PhiX genome (accession NC_001422.1) using Bowtie2 v.2.4.2 (Langmead and Salzberg, 2012). PCR primers were trimmed with CUTADAPT v.3.4 (Martin, 2011) allowing one mismatch. Paired-end reads were merged using the *fastq_mergepairs* function in VSEARCH, followed by filtering low quality reads using the *fastq_filter* function in VSEARCH allowing a maximum expected error of one (Edgar and Flyvbjerg, 2015). Dereplication was performed using the *derep_fulllength* function in VSEARCH and followed by delineation of amplicon sequence variants (ASVs; Callahan et al., 2017) using the UNOISE algorithm implemented in VSEARCH with the *cluster_unoise* function (Edgar, 2016b) using an alpha of two and a minsize of eight. Identification and removal of potentially chimeric ASV sequences was performed using the UCHIME2 algorithm *via* the *uchime3_denovo* function (Edgar et al., 2011) in VSEARCH. For the remaining sequences, target verification was conducted using Metaxa2 v.2.2.0 (Bengtsson-Palme et al., 2015) for the 16S rRNA gene (bacteria/archaea) and ITSx v1.1.3 (Bengtsson-Palme et al., 2013) for the ITS region (fungi), whereas unverified sequences were discarded. The quality filtered reads were run against the verified ASV sequences using the *usearch_global* algorithm with settings maxrejects at 32, maxaccepts at 0, maxhits at 1, and a minimum identity threshold at 97%. Each verified ASV sequence was taxonomically classified by running the Sintax algorithm (Edgar, 2016a) against the SILVA v.132 database (Pruesse et al., 2007) for the 16S rRNA gene sequences and the UNITE database v8.0 (Nilsson et al., 2019) for the ITS region at a confidence cutoff of 0.8. ASVs not assigned to bacteria, archaea, and fungi, or assigned to organelle structures (chloroplasts, mitochondria) were removed.

### Statistics

Statistical analyses were conducted in R version 4.0.5 (R Core Team, 2021) and the R script is provided in the Supplementary Material. Differences in physicochemical soil characteristics, DWY, and basal respiration between treatments within timepoints (incorporating block as an additional factor) was assessed using ANOVA. Subsequently, differences between factor levels were analyzed with Tukey’s HSD test. Normality of residuals and homoscedasticity was confirmed with the Shapiro–Wilk test and standardized residuals plots, respectively. If these assumptions were not met, the Friedman Rank Sum Test was used analogously to assess the significance of factors, whereas the Conover post-hoc test from the PMCMR package v.4.4 (Pohlert, 2014) was used as post-hoc test for multiple comparisons between factor levels, adjusting *p*-values with the Bonferroni method. Correlations among these variables were calculated using the squared Pearson’s correlation coefficient (R^2^).

The following depicted operations were largely based on functions from the *vegan* package v.2.5–7 (Oksanen et al., 2020) in R. An iterative (100 iterations) subsampling approach of the ASV matrices (Martiny et al., 2017; Hemkemeyer et al., 2019) was applied to remove systematic biases in read counts potentially arising from the sequencing workflow (McKnight et al., 2019). Alpha-diversity was assessed by calculating observed richness, Shannon diversity index and Pielou’s evenness of ASVs (Chao et al., 2014) based on the median of 100-fold subsampled ASV matrices using the functions *rarefy*, *specnumber*, and *diversity* in *vegan*. Beta-diversity was assessed based on the median Bray–Curtis dissimilarity calculated from the 100-fold subsampled ASV-matrices using the function *vegdist* in *vegan* and followed by a step-wise approach laid out by Anderson and Willis (Anderson and Willis, 2003). Briefly, first major variance components were identified by a principal coordinate analysis (PCoA, Gower, 2015) by the function *cmdscale*. Relationships between soil physicochemical properties and microbial beta-diversity were assessed by creating join-biplot PCoAs using the function *envfit* in *vegan*. Treatment effects were assessed using a multivariate permutational analysis of variance (PERMANOVA, Anderson, 2001) implemented as the *adonis2* function in *vegan*. Since significant differences detected by PERMANOVA can not only arise from differences in means but also from differences in dispersion (analogous to heteroscedasticity in a regular ANOVA), homogeneity of variance was checked using permutational analysis of multivariate dispersion (PERMDISP, Anderson, 2006) implemented as the *betadisper* function in *vegan*. *p*-values of pairwise PERMANOVA tests were corrected for multiple testing using the Benjamini-Hochberg method (Benjamini and Hochberg, 1995) implemented in the *p.adjust* function. Finally, beta-diversity was constrained by the significant factors using canonical analysis of principal coordinates (CAP, Anderson and Willis, 2003) implemented as the CAPdiscrim function in the R package *BiodiversityR* v2.14–2 (Kindt and Coe, 2005). PERMANOVA, PERMDISP, CAP were run with 999 permutations. In order to assess the dissimilarity between fertilizer and no-fertilizer treatments, we calculated the mean Bray-Curtis dissimilarities of one replicate of the fertilizer treatments to the five replicates of the no-fertilizer treatment at the same sampling day.

Differences of individual ASVs across treatments was assessed using univariate PERMANOVA and PERMDISP on the median of 100-fold subsampled ASV matrices. Type I error inflation due to multiple testing was controlled by the false discovery rate correction approach according to Storey (2002) using the package *qvalue* v.2.22.0 (Storey et al., 2020). ASVs were classified as “sensitive” to treatment if (I.) PERMANOVA results were significant and (II.) significance did not arise from significant dispersion (PERMDISP). The analysis of sensitive ASVs was focused on the soil microbial data of sampling day 42 only to reduce the amount of confounding extracellular DNA potentially imported with fertilizers, which is further addressed in the discussion section. Additionally, to reduce noise from rare ASVs (low read counts across all samples) for the analysis of sensitive ASVs, prefiltering was applied. ASVs with a minimum relative abundance of 0.015% (rare) were removed. For all statistical tests, significance was accepted at *p* < 0.05 or *q* < 0.05, if applicable.

## Results

### Physicochemical characteristics of fertilizers and fertilizer-amended soils

BR tended to be drier and contain more C, N, NH_4_^+^, and P than CC (Table 2). BR also showed higher EC and was closer to a neutral pH compared to CC. CC in turn tended to show higher contents of the remaining elements.

**Table 2:** Physicochemical characteristics of the applied fertilizers.

Parameter^1^	BR ^2^	CC
DM [%]	87.4^3^	63.4
NH_4_^+^-N [mg kg^−1^]	4,667.3	34.8
NO_3_^−^ -N [mg kg^−1^]	250.2	181.4
EC [μS cm^−1^]	1,236	772
pH	6.9	7.5
N [g kg^−1^]	31.6 ± 1.3	15.0 ± 1.1
C [g kg^−1^]	400.7 ± 9	173.5 ± 9.5
C:N ratio	12.7 ± 0.6	11.6 ± 0.3
P [g kg^−1^]	5.6 ± 0.3	2.4 ± 0.2
K [g kg^−1^]	2.7 ± 0.1	3.0 ± 0.2
Ca [g kg^−1^]	6.4 ± 0.4	23.7 ± 3.8
Mg [g kg^−1^]	2.2 ± 0.1	3.2 ± 0.5
Na [g kg^−1^]	0.2 ± 0.0	0.5 ± 0.0
Fe [g kg^−1^]	4.1 ± 1.0	21.0 ± 6.4
Mn [g kg^−1^]	0.1 ± 0.0	0.9 ± 0.1
Zn [g kg^−1^]	0.1 ± 0.0	0.1 ± 0.0
Al [g kg^−1^]	3.6 ± 0.9	13.0 ± 0.5
Cu [mg kg^−1^]	12.8 ± 1.3	28.6 ± 5.2
Cd [mg kg^−1^]	ND^4^	ND

1 Ammonium-N (NH_4_^+^-N), nitrate-N (NO_3_^−^-N), electrical conductivity (EC), pH (in H_2_O), N (total nitrogen)_,_ C (total carbon), C:N ratio (ratio between C and N), P (total phosphorous)_,_ K (total potassium), Ca (total calcium)_,_ Mg (total magnesium), Na (total sodium)_,_ Fe (total iron), Mn (total manganese), Zn (total zinc), Al (total aluminum), Cu (total copper), CD (total cadmium).

2 Values are based on dry weight (average ± standard deviation if measured in technical replicates, *n* = 3).

3 The first five properties could not have been measured in triplicates since they require larger quantities of dried material and only small quantities of the fertilizers were received.

4 ND, below detection limit.

**Figure 1:**
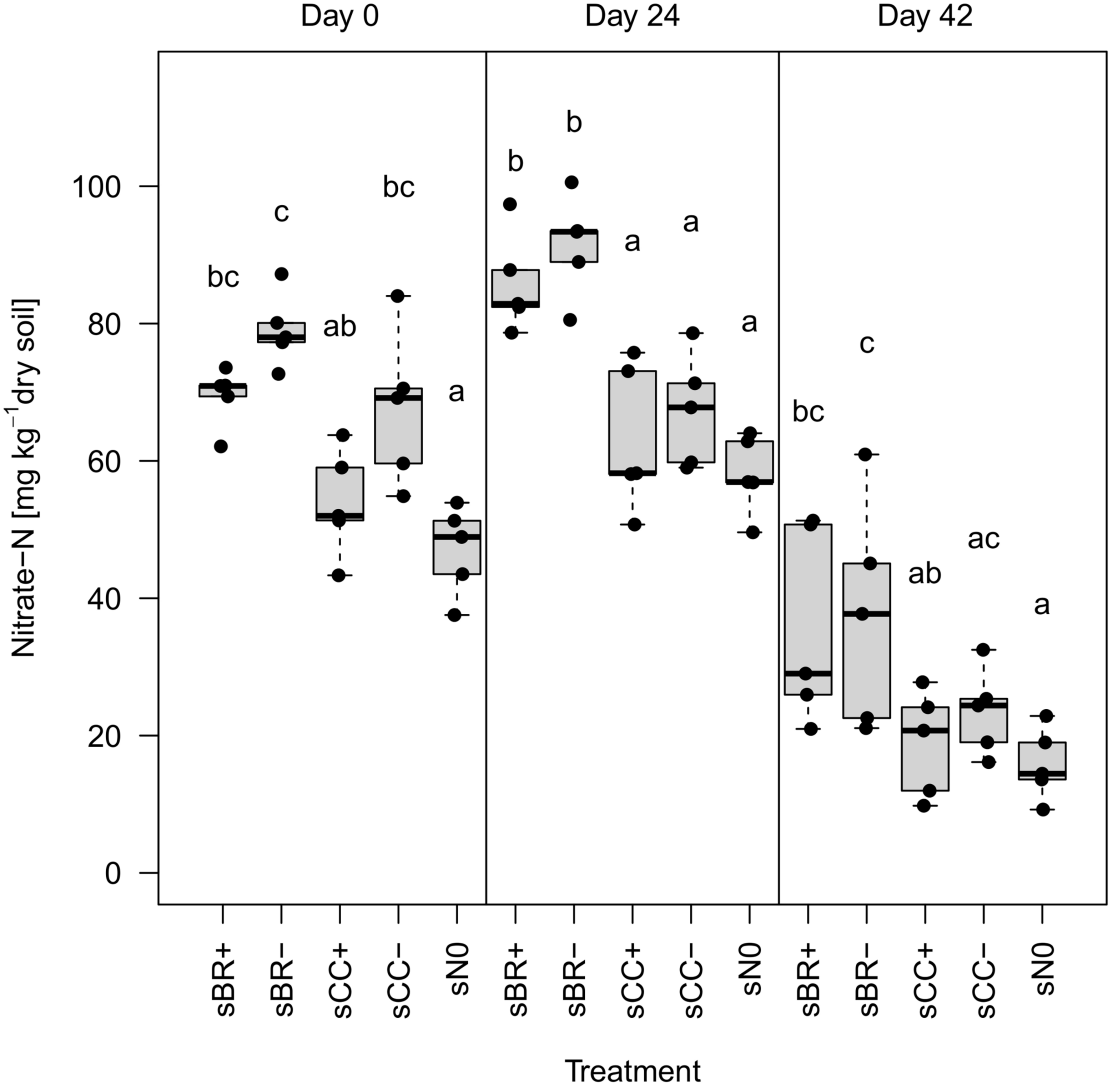
Nitrate-N (NO_3_^−^-N) content in the different fertilizer-amended soils and in the no-fertilizer treatment. Distinct lower-case letters indicate significant (*p* < 0.05, n = 5) differences among treatments for the same sampling date. sBR+, soil fertilized with BSFL residue; sBR-, soil fertilized with sterile BSFL residue; sCC+, soil fertilized with conventional compost; sCC-, soil fertilized with sterile conventional compost; sN0, no-fertilizer treatment.

Overall, application of fertilizers had a minor influence on soil physicochemical parameters (Supplementary Tables 1, 2). However, BR fertilizers significantly (*p* < 0.05) increased NO_3_^−^-N content in soil over all sampling dates compared to sN0 (Figure 1). On day 24 post application, BR fertilizers significantly (*p* < 0.05) increased NO_3_^−^-N also compared to CC fertilizers. Furthermore, NO_3_^−^-N in soil generally dropped by more than half from day 24 to 42. Soil NH_4_^+^-N contents did not show this pattern and accounted for only around 2% (1.12 ± 0.89 mg NH_4_^+^ kg^−1^ soil) of the total mineral N. EC values strongly correlated with NO_3_^−^-N contents (R^2^ = 0.85, *p* < 0.001) and showed a similar significance pattern across the treatments (Supplementary Table 2). GWC varied among sampling timepoints only. Significant differences in pH, as well as C and N contents among treatments and over time occurred only erratically and lacked trends.

### Microbial community composition in fertilizers

**Figure 2:**
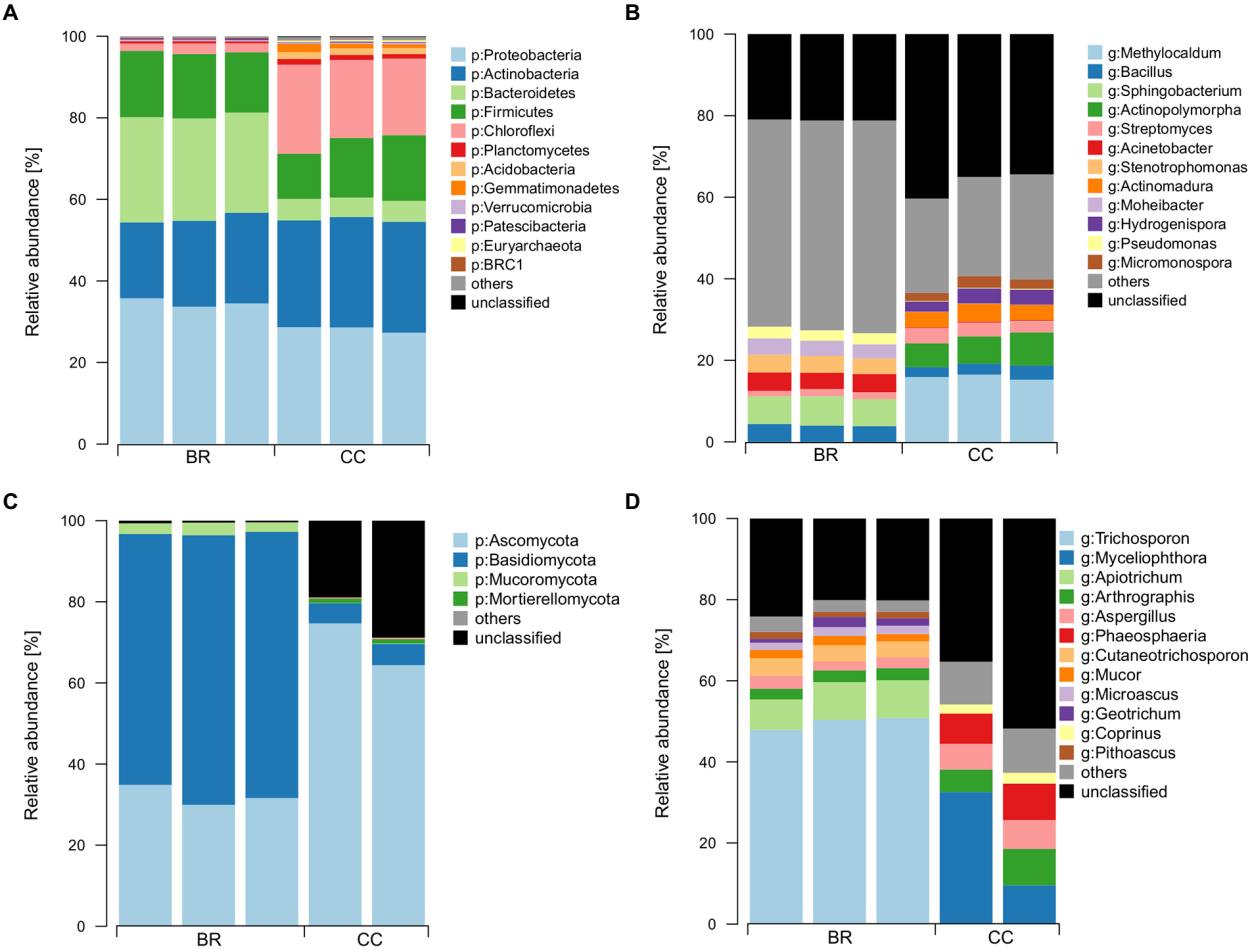
Most abundant bacterial and fungal phyla (**A** and **C**, respectively) and genera (**B** and **D**, respectively) in the two organic fertilizers. BR: BSFL residue, CC: conventional compost.

Sequencing yielded 326,647 (54,441 ± 4,680 per replicate) bacterial/archaeal and 176,527 (35,305 ± 7,100 per replicate) fungal reads delineated into 4,037 and 383 ASVs, respectively. For fungi, one replicate of CC had to be excluded from the analysis for showing only 56 read counts. Fertilizers differed in bacterial/archaeal taxonomic composition (Figures 2A,B). At phylum level, Proteobacteria (31%), Actinobacteria (24%), Bacteroidetes (15%), Firmicutes (15%), and Chloroflexi (11%) were the most abundant groups, whereas Chloroflexi was proportionally more abundant in CC (20%) than in BR (2%), at the expense of Bacteroidetes (5% in CC, 25% in BR). At genus level, taxonomic composition differed strongly, with only *Bacillus* (3%) and *Streptomyces* (2%) among the predominant genera (>0.5%) shared between CC and BR (Figure 2B).

Ascomycota and Basidiomycota were the predominant fungal phyla in the fertilizers, with Basidiomycota showing much higher relative abundance in BR (>64%) than CC (5%; Figure 2C). Interestingly, a large fraction (up to 28%) of the sequences in CC could not be assigned at phylum level, whereas this fraction was small (<1%) in BR. Again, only very few of the abundant genera were shared between the fertilizers, e.g., *Arthrographis* (5%) and *Aspergillus* (4%, Figure 2D).

For both domains but particularly for bacteria/archaea, CC tended to be richer in ASVs (bacteria: BR:1632 ± 7, CC: 2022 ± 78; fungi: BR:171 ± 4, CC:184 ± 10). For bacteria, Pielou’s evenness tended to be higher in BR (BR:0.84 ± 0.00, CC:0.72 ± 0.00), while this trend did not apply to fungi (BR:0.52 ± 0.01, CC:0.6 ± 0.08). BR showed higher bacterial/archaeal (6.23 ± 0.03) and lower fungal Shannon diversity (2.66 ± 0.07) when compared to CC (5.49 ± 0.03 and 3.14 ± 0.44, respectively).

### Impact of fertilizers on soil microbial community

#### Soil microbial diversity

The bacterial/archaeal community in the initial unamended soil (sN0 at day 0, Supplementary Figure 2) was dominated by Proteobacteria (26%), Actinobacteria (23%) and Acidobacteria (16%) and the most abundant genera included *Candidatus Xiphinematobacter* (2%), *Bacillus* (2%), *Gaiella* (2%), and *Candidatus Udaeobacter* (2%). All taxa assigned to the domain archaea (2%) belonged to the family *Nitrososphaeraceae* (2%) and were not further classified. The fungal community was dominated by Ascomycota (68%) followed by Mortierellomycota (14%), and Basidiomycota (9%) and the most abundant genera included *Mortierella* (10%), *Fusarium* (3%), *Staphylotrichum* (3%), *Lecythophora* (3%).

**Figure 3:**
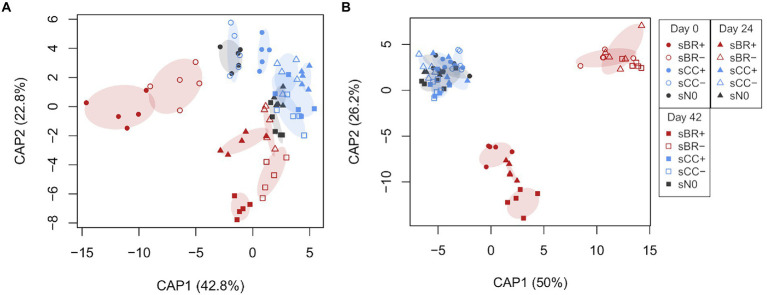
Constrained analysis of principal coordinates (CAP) ordinations elucidating *a priori* differences in bacterial/archaeal **(A)** and fungal **(B)** community structure upon fertilizer amendment at days 0, 24, 42 of the experiment. Percent between group variation represented by each canonical axis are provided in parentheses next to the axis headers. CAP reclassification success rates for bacteria/archaea at day 0: sBR+, 100%; sBR-, 100%; sCC+, 100%; sCC-, 60%; sN0, 80%; day 24: sBR+, 80%; sBR-, 80%; sCC+, 100%; sCC-, 40%; sN0, 80%; day 42: sBR+, 100%; sBR-, 100%; sCC+, 40%; sCC-, 60%; sN0, 80%. CAP reclassification success rates for fungi at day 0: sBR+, 80%; sBR-, 100%; sCC+, 0%; sCC-, 20%; sN0, 60%; day 24: sBR+, 100%; sBR-, 100%; sCC+, 0%; sCC-, 60%; sN0, 80%; day 42: sBR+, 100%; sBR-, 100%; sCC+, 40%; sCC-, 40%; sN0, 0%. sBR+, soil fertilized with BSFL residue; sBR-, soil fertilized with sterile BSFL residue; sCC+, soil fertilized with conventional compost; sCC-, soil fertilized with sterile conventional compost; sN0, no-fertilizer treatment.

The addition of fertilizer was a significant (PERMANOVA, *p* < 0.001) driver of soil microbial community structure (Table 3). The impact on soil fungi was more pronounced compared to the impact on soil bacteria/archaea (Figure 3; Supplementary Figure 3). Fertilization explained bacterial/archaeal and fungal variation in soil community structures by on average 7 and 21%, respectively. Application of BR+ led to soil microbial communities significantly (PERMANOVA, *p* ≤ 0.002) distinct from soil amended with BR- and unfertilized soil. However, significant differences between bacterial/archaeal communities among fertilizer treatments were not only arising from dissimilarity but also from dispersion.

**Table 3:** Effects of fertilizer treatment on microbial beta-diversity determined by PERMANOVA. Significant values (*p* < 0.05) are indicated in bold.

*Test* *^1^*	Bacteria & Archaea					Fungi				
	F	P	R^2^	F	P	R^2^
Treatment	1.5	**0.001**_**SD**_	0.07	5.1	**0.001**	0.21
Sampling day	1.7	**0.001**	0.04	2.4	**0.001**	0.05
Treatment x Sampling day	1.2	**0.001**_**SD**_	0.12	1.6	**0.001**	0.13
**Treatments** *^2^*	**sBR+**	**sBR−**	**sCC+**	**sCC−**		**sBR+**	**sBR−**	**sCC+**	**sCC−**	
sBR-	**0.002**					**0.001**				
sCC+	**0.001**	**0.001**				**0.001**	**0.001**			
sCC-	**0.001**	**0.002**	**0.028**			**0.001**	**0.001**	0.221		
sN0	**0.001**	**0.001**	**0.001**	**0.001**		**0.001**	**0.001**	0.552	0.075	

1 Effects of factors and their interaction were analyzed by permutational analysis of variance (PERMANOVA). Factors are fertilizer treatment (degrees of freedom = 4, sBR+, soil fertilized with BSFL residue; sBR-, soil fertilized with sterile BSFL residue; sCC+, soil fertilized with conventional compost; sCC-, soil fertilized with sterile conventional compost; sN0, no-fertilizer treatment soil), day of sampling (degrees of freedom = 2, day 0, 24, 42), and their interaction. Values represent the pseudo-F ratio (F) and the level of significance (P). Values at *p* < 0.05 are shown in bold. SD indicates significant dispersion.

2 *P*-values of pairwise comparisons between fertilizer treatments with P-values adjusted for multiple comparisons using the Benjamini–Hochberg method. Values at *p* < 0.05 are shown in bold.

BR+ appeared to introduce stronger effects to the microbial community than CC+ (Figure 3), but notably for bacteria/archaea both pairwise comparisons with sN0 were significant (*p* < 0.05). For bacteria/archaea differences between the influence of BR+ and CC+ became most apparent at sampling days 0 and 42 based on the dissimilarity to sN0 (Supplementary Figure 4). In general, BR fertilizers tended to have a more clear-cut effect on the soil fungal community than the composts.

Time had a significant (PERMANOVA, *p* < 0.001) impact on bacterial/archaeal and fungal communities in soil (Table 3; Figure 3). The sampling day explained on average 4 and 5% of their variation, respectively. Bacterial/archaeal communities were significantly (*p* < 0.001) changing between each sampling day. In contrast, only the fungal community at day 0 was significantly distinct from the later sampling days 24 and 42 (*p* = 0.028 and *p* = 0.006, respectively). The interaction between sampling day and treatment had a significant (*p* ≤ 0.001) impact on bacterial/archaeal and fungal communities and accounted for on average 12 and 13% of the variation, respectively.

Comparing among treatments, physicochemical soil properties C, N, C:N and pH minorly affected soil microbial communities. NO_3_^−^-N content and EC were the only soil properties significantly correlated with bacterial/archaeal (*p* = 0.004 and *p* = 0.004, respectively) and fungal (*p* = 0.014 and *p* = 0.025, respectively) beta-diversity patterns. Along the gradient of NO_3_^−^-N bacterial/archaeal communities were lining up by timepoints whereas fungal communities were rather lining up by fertilizer types (Supplementary Figure 5).

#### Sensitive taxa

The application of BSFL residues significantly (*q* < 0.05) impacted the soil microbiome on individual ASV levels. A total of 42 fungal and 16 bacterial/archaeal ASVs were identified as sensitive to treatments (Supplementary Tables 5, 6) at day 42. A comparable number of fungal and bacterial/archaeal ASVs (11 and 12 respectively) were enriched under BR+ treatment. Nevertheless, the total relative abundance of sensitive ASVs assigned to fungi and enriched under BR+ was around five times higher than for bacteria. ASVs thriving under BR fertilizers in general (BR+ and BR-) were among others assigned to the fungal genus *Mortierella* and the family *Lasiosphaeriaceae*. However, under BR+ treatment only ASVs assigned to the genera *Mucor*, *Cephaliophora* and a not further classified fungus were identified as enriched. With respect to bacteria /archaea just one ASV assigned to bacterial family *Bacillaceae* was thriving under BR fertilizers in general. Bacterial /archaeal ASVs thriving under BR+ only were among others assigned to the bacterial genus *Bacillus*, the family *Chitinophagaceae* as well as to the archaeal family *Nitrososphaeraceae* (Supplementary Tables 5, 6). Few fungal and bacterial/archaeal ASVs that were promoted in soil by BR+ application were also identified in BR itself hinting to potential introduction. These ASVs were assigned to the fungal genera *Mucor, Botryotrichum, Cephaliophora,* and *Mortierella* and to the bacterial genus *Bacillus,* family *Bacillaceae* and order *RBG-13-54-9*.

### Impact of fertilizers on basal respiration

**Figure 4:**
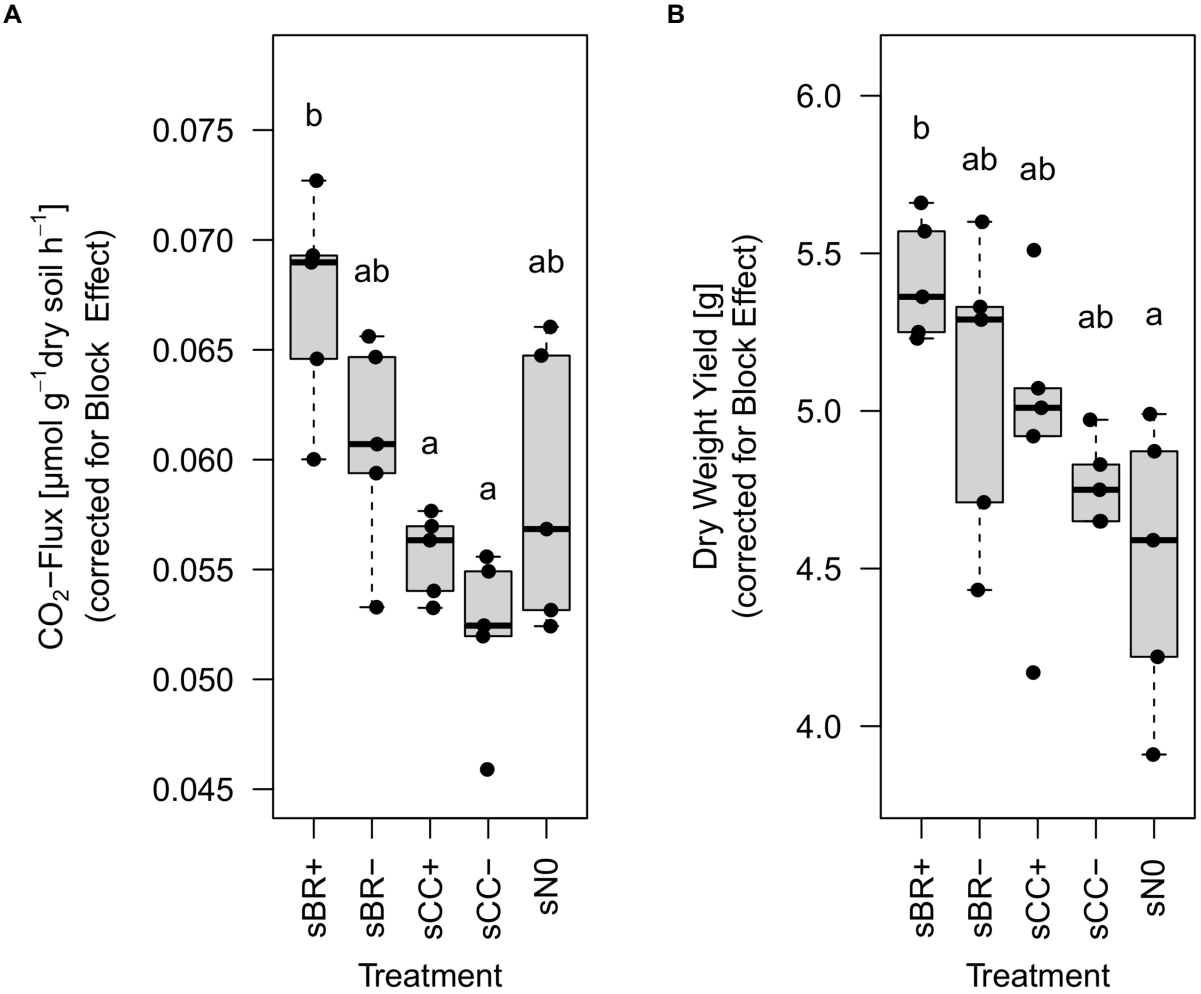
Basal respiration **(A)** and grass-clover dry weight yield **(B)** at the end of the experiment (day 42) from the different fertilizer amended soils. Distinct lower-case letters indicate significant (*p* < 0.05), n = 5 differences among treatments as determined by Tukey’s HSD test, values were corrected for the significant block effect as determined by ANOVA. sBR+, soil fertilized with BSFL residue; sBR-, soil fertilized with sterile BSFL residue; sCC+, soil fertilized with conventional compost; sCC-, soil fertilized with sterile conventional compost; sN0, no-fertilizer treatment.

Regression models fitted well to the development of CO_2_ concentrations over time in the headspace of incubated soil samples with an R^2^ of 0.97 ± 0.02 over all experimental units. At the end of the experiment, soil fertilized with BR+ emitted significantly (*p* < 0.02) more CO_2_ than soil treated with CC fertilizers whereas BR- showed values in between (Figure 4A; Supplementary Table 2). BR+ amended soil emitted around 20% (≈0.01 μmol g^−1^ h^−1^) more CO_2_ than CC+ and CC- amended treatments (Supplementary Table 2). Even though BR+ treated soils showed the highest respiration values, these were not statistically different to BR-amended or unamended soils (sN0). The positive correlation between basal respiration (corrected for block effect) and NO_3_^−^-N at day 42 was significant but weak (R^2^ = 0.22, *p* = 0.018).

### Impact of fertilizers on yield

At the end of the pot experiment, plants growing on soil fertilized with BR+ had around 17% more (*p* < 0.05) biomass than the no-fertilizer treatment (Figure 4B; Supplementary Table 2). However, even though sBR+ showed the highest mean values, no significant difference was found compared to the other fertilization treatments. Only on sampling day 24 there was a significant but weak correlation between DWY and NO_3_^−^-N observable (R^2^ = 0.14, *p* = 0.036).

## Discussion

### Microbial taxa associated with BSFL guts and residues

The recurrence of certain microbial taxa in BSFL residues is especially interesting for the assessment of their reliability in introducing BSFL- and soil fertility-related microbes to the soil. Therefore, taxa found in BR are contrasted with those that are commonly found to be associated with BSFL residues or BSFL themselves in literature.

#### Bacteria/Archaea

Firmicutes, Proteobacteria, and Bacteroidetes were identified as characteristic for guts and residues of BSFL (Gold et al., 2020b). Those were three of the four most abundant bacterial phyla we have found in BR. Especially Bacteroidetes were strongly enriched in BR compared to CC (Figure 2). Members of the bacterial phylum Bacteroidetes are important animal symbionts and decomposers of high molecular weight organic matter (Thomas et al., 2011). Members of the proteobacterial genus *Pseudomonas* and the actinobacterial genus *Actinomyces* were also enriched in BR. These genera were reported to be characteristic for the gut of the larva (Klammsteiner et al., 2020b). Other genera recurringly associated with the gut and residues of BSFL like *Dysgonomonas*, *Morganella*, *Providencia*, and *Proteus* (Gold et al., 2020b) were not present in BR or only in low abundances (<1%). The BSFL residues used in this study had a high DM content (87.4%) compared to average reported values for BSFL residues of around 69% (values ranging between 40 and 90%; Gärttling and Schulz, 2021). Thus, BR might have resembled less the conditions of insect guts compared to moister residues, which are based on distinct substrates or rearing conditions. That could have limited the maintenance of gut-associated taxa in BR.

*Sphingobacterium, Pseudomonas and Bacillus* were predominant bacterial genera in BR, that were also previously identified in BSFL residues from brewery waste and fruits summarized by Gold et al. (2020b) from Wynants et al. (2019). These three genera were also identified in mealworm residues and are known plant-associated taxa potentially involved in plant growth promotion (Poveda et al., 2019). Members of *Sphingobacterium* were reported to play a major role in the decomposition of complex organic matter, whereas *Pseudomonas* and *Bacillus* were associated with the control of plant pathogens (Lutz et al., 2020; Scotti et al., 2020).

#### Fungi

Compared to bacteria little data is available on fungi associated with BSFL. One study suggests that the fungal community found in BSFL guts is mainly shaped by the diet (Boccazzi et al., 2017), which lowers the probability of a core mycobiome to be passed on from BSFL to residues. The study by Tanga et al. (2021) proposed *Pichia* as a prevalent genus in BSFL guts across different diets including brewery waste. Furthermore, Kuznetsova et al. (2021) reported that *Pichia* and *Diutina* dominated (99.9%) the residues after rearing BSFL for 5 days on food waste. We found *Pichia* to be present in BR, but only in low abundances (<0.1%). The high relative abundance of Basidiomycota in BR compared to CC can almost exclusively be attributed to members of the most abundant genus *Trichosporon*, which has been identified previously in BSFL guts (Tanga et al., 2021) and as the dominant basidiomycetous genus in substrates exposed to BSFL (Bernard et al., 2020). Indeed, BSFL were suggested to associate with the genus to suppress pathogenic bacteria in the rearing environment of the larvae (Gorrens et al., 2021). *Trichosporon* has been reported to degrade aromatic compounds such as phenols (Middelhoven, 1993; DeRito and Madsen, 2009). In fact, the high abundance of *Trichosporon* in BR might be an indicator of elevated concentrations of phenolic and thus aromatic substances, the chemical compounds of lignin. Interestingly, Song et al. (2021) related increased concentrations of phenolic substances in uncomposted BSFL residues to hampered plant performance in comparison to their composted analogue. BR did not undergo a composting process. However, effects of phytotoxicity were not observable in our experiment. Phytotoxicity might anyway only become apparent upon direct comparison between composted and uncomposted residues applied at higher rates.

### Changes in soil microbial communities upon fertilization with BSFL residues

Extracellular DNA derived from dead cells is a well described and potentially confounding factor in microbial community analysis (Carini et al., 2016; Lennon et al., 2018). Sirois and Buckley (2019) reported >99% degradation of extracellular DNA (eDNA) in soils within 7 days irrespective of the moisture content, temperature and tillage of the soil. However, a small amount of eDNA with little potential to bias beta-diversity analysis remained determinable throughout their trials (39–80 days). Consequently, an artifact arising from eDNA imported with sterile fertilizer especially at sampling day 0 cannot be ruled out. To minimize the impact of eDNA on our individual ASV results, the analysis of sensitive ASVs was conducted on the last sampling (day 42). Furthermore, soil conditions were expected here to be closest to an assumed “steady state” including thorough root permeation throughout the soil profile.

This study aimed to examine the impact of BSFL residues on the plant-associated soil microbiome. We showed that BSFL residues can shape the soil microbial community and to a larger extent than composts. Results from Chiam et al. (2021) suggest the same finding for bacteria only and using much higher fertilization rates (30% v/v BSFL residue or compost +70% v/v soil). By sterilizing fertilizers prior to their application, we sought to disentangle the impact of biotic and abiotic drivers of BR on the soil microbial community. Both biotic and abiotic components appeared to influence the soil microbial community structure in general. However, on the soil bacterial/archaeal community, biotic factors appeared to have a stronger effect, since the unsterilized BR+ introduced stronger changes than the sterilized counterpart BR- (Figure 3; Supplementary Figure 4). Furthermore, differences in the bacterial/archaeal community structure were not as clear-cut as in the fungal community structure since dispersion influenced the significance of our results (Table 3).

#### Influence of the abiotic part of BSFL residues

We propose the addition of organic matter to the soil as a major abiotic driver of the observed microbial patterns. Gärttling and Schulz (2021) showed that BSFL residues comprise high contents of organic matter of around 86% and can thus be an important energy source for soil microbes. However, in our experiment, total soil C as a proxy for organic matter was not significantly influenced by the fertilization treatments. Nevertheless, BR fertilizers had a stronger impact on soil microbial communities than the compost (Figure 3; Supplementary Figure 4). Thus, probably rather the type than the amount of organic matter that was added was decisive. On one hand, the organic C in CC was highly stabilized over the six-month composting process (Garcia et al., 1991) and was thereby less available to microbes. On the other hand, BSFL residues used in our experiment were produced by BSFL feeding on brewer’s spent grain. Roth et al. (2019) report that brewer’s spent grain consists of up to 70% fiber (cellulose, hemicellulose and lignin), whereas the hardly degradable lignin alone may be enriched by up to 28% in the organic waste. Reduction of cellulose and hemicellulose by BSFL treatment is highly variable but may account for up to 50%, whereas lignin reduction may account for up to 2% if any (Gold et al., 2018). Thus, the residue used in our experiment was probably high in fiber and especially in lignin. As discussed above, the high abundance of the fungal genus *Trichosporon* in the residue supports this assumption. These complex organic compounds are known to be mainly degraded by fungi, whereas bacteria use the more labile C sources (Insam and De Bertoldi, 2007; Kirchman, 2018). The fact that also sterile BR fertilizers were strongly influencing soil fungal diversity might thus have been a result of fiber addition. This is supported by the fact that taxa from the fungal species *Mucor circinelloides,* genus *Mortierella* and family *Lasiosphaeriaceae* were enriched under BR fertilizer application in general. Members of the taxa have been reported for decomposing cellulose (Saha, 2004), hemicellulose (Jackson, 1965) and wood (Nordén and Paltto, 2001), a fiber-rich organic matter, respectively. Our discussion emphasizes that the type of organic waste BSFL residues are originating from is likely influencing the soil microbiome when applied as fertilizer. Whether our results are generalizable might therefore depend on a generally high fiber content in BSFL residues compared to other organic fertilizers, which to our knowledge has not yet been assessed. The assumption is supported by recurrently highlighted stimulations of soil fungi after application of BSFL residues (Temple et al., 2013; Rummel et al., 2021; Watson et al., 2021).

The presence of chitin, a more labile form of organic matter, in BSFL residues might have also contributed to a shift in the soil microbial community. Gebremikael et al. (2020) suggested chitin as a major abiotic driver of change in microbial communities fertilized with BSFL residues. Due to BSFL shedding their exoskeletons during rearing (Gold et al., 2018) chitin and its derivates are an integral part of the residue. Crocker et al. (2019) reported impacts on the soil microbial community structure when pure chitin was added to the soil. Previous studies indicated effects of chitin contained in BSFL residues on the soil microbiome. In fact, the content of chitin or its derivates in BSFL residues from different origins is still to be assessed. However, Panov (2021) showed that chitinolytic organisms and chitinase activity were promoted when BSFL residues were added to soil even in low quantities (1.6% v/v). Gebremikael et al. (2022) found increased chitinase activity in soil fertilized with BSFL residues produced on distinct organic wastes (0.11 g N kg^−1^ DW soil) over the duration of their 103-day incubation experiment. Notably chitinase activity was not only enhanced compared to the control soil but also to soil treated with a distinct organic fertilizer. In our experiment *Mortierella,* a fungal genus associated with strong chitinolytic capabilities (Jackson, 1965; Okafor, 1966; Gray and Baxby, 1968), was enriched in soils treated with BR+ and BR-. That finding supports the assumption that chitin in the residues was impacting the soil microbial community. What speaks against a strong effect of chitin on soil microbes on community level is the limited response of bacteria/archaea in soils fertilized with BR-. Since bacteria generally play a key role in chitin degradation (Beier and Bertilsson, 2013), a chitin-enriched BR- would probably have led to a more pronounced shift in their community composition. The presence and bioavailability of chitin in BSFL residues should undergo further scrutiny. The molecule and its derivates are considered promising substances for agricultural management (Sharp, 2013). Among others they can be used to control plant pathogens like nematodes and pathogenic fungi. Stimulation of chitin degrading antagonists is one proposed underlying mechanism (Yang and Fukamizo, 2019). Former studies suggested a link between the chitin inherent to BSFL residues and the suppression of soil-borne crop diseases (Gebremikael et al., 2020; Quilliam et al., 2020). However, the disease suppressiveness of BSFL residues is not always given (Elissen et al., 2019). And different ways of post-processing such as composting of the residues might reduce the content of the easily degradable chitin (Insam and De Bertoldi, 2007; Oberlintner et al., 2021).

The physicochemical properties that were measured in the fertilizers and soils appeared to have little influence on differences in soil microbial community structure. Correlations of physicochemical variables onto the community ordinations were only significant for soil NO_3_^−^ -N content and EC. EC was highly correlated to NO_3_^−^-N and thus probably mainly driven by this plant nutrient as observed before (Blume et al., 2010). Indeed, due to an already nutrient-rich experimental soil, the addition of fertilizers had a limited effect on the physicochemical variables (Supplementary Table 2), except for NO_3_^−^-N (Figure 1). Long-term mineral N addition may influence microbial diversity in soil by favoring microbes with tolerance to high osmotic potential (Wang et al., 2018). However, the experimental period was short and the increase in NO_3_^−^ mediated by addition of BR fertilizers was relatively moderate. BSFL residues are expected to have larger effects on the soil microbial community when applied in nutrient poor agricultural systems.

#### Influence of the biotic part of BSFL residues

Gebremikael et al. (2020) postulated that the microbiome inherent to insect residues can, similarly as suggested for other organic soil amendments (Sun et al., 2016), impact the soil microbial community when used as a fertilizer. We could show that the biota inherent to BSFL residues can significantly influence the rhizosphere soil microbial community, since differences between soil fertilized with untreated and sterile BSFL residues were significant (Table 3). This effect might stem directly from microbes present in BR+ that were introduced to the soil. We showed that at the end of our experiment few microbes significantly enriched in soil fertilized with BR+ were also identified in the fertilizer itself (Supplementary Tables 5, 6) e.g. ASVs linked to the fungal species *Cephaliophora tropica* and *Mucor circinelloides* or the bacterial genus *Bacillus*. As mentioned, *Bacillus* has been identified in BSFL residues (Gold et al., 2020b) and insect residues in general and was linked to their positive effects on crops (Barragán-Fonseca et al., 2022). Notably, also members of the beforementioned genus *Trichosporon,* that dominated BSFL residues, were only highly abundant in BR+ amended soil. However, those ASVs assigned to *Trichosporon* did not qualify for being sensitive taxa due to significant inhomogeneity of variance (dispersion). Nevertheless, this taxon should further be observed as a potential candidate that is transferred from BSFL residues to the soil. Going one step further, results from our setting give little indication that BSFL residues can act as a vector for microbes associated with the larvae themselves to compete in the rhizosphere, as it has been implied for insect residue (Poveda, 2021).

We assume that microbes, that were potentially introduced *via* BSFL residues, contributed also indirectly to the differences in microbial communities between soil fertilized with BR+ and BR-. In fact, especially bacterial/archaeal ASVs significantly enriched under BR+ alone were rarely identified in the fertilizer itself (Supplementary Table 5). It is possible that these taxa enriched under BR+ were promoted by metabolic products from introduced taxa. For instance, decomposers introduced with BR+ could have made nutrients and energy sources available to depending bacteria/archaea (Beare et al., 1997). Indeed, members of the archaeal family *Nitrososphaeraceae* were enriched under BR+ fertilization only. They are known ammonia-oxidizing organisms that rely on the provision of NH_4_^+^ and may use organic compounds as carbon sources (Tourna et al., 2011; Kerou and Schleper, 2015). Both are principal sources that arise from microbial mineralization of organic matter. Beyond that it is possible that organisms distinct from fungi or bacteria/archaea were introduced to the soil *via* BSFL residues and affected the soil microbial community by their metabolites. Little is known about the meso-and microfauna of BSFL residues, but it is probable that also here they are involved in organic matter decomposition as much as they are generally in composts and soils (Blume et al., 2010; Steel and Bert, 2012) and would therefore also be relevant to the biotic and abiotic environment of the soil they are introduced to.

It has to be noted that the fertilizer sterilization could have contributed to a change in the soil microbial community. HEEB, a non-thermal sterilization method, can be considered as a relatively gentle procedure in comparison to thermal sterilization. However, it cannot be ruled out that HEEB led to changes in the bioavailability of nutrients in fertilizers.

### BSFL residues and soil fertility

Our study sought to further identify whether BSFL residues can contribute to soil fertility *via* an impact on the soil microbiome. Soil microbial diversity has previously been associated with soil fertility (Mäder et al., 2002), however a change in the microbial community composition does not automatically translate into a change in their performance due to functional redundancy among microbes (Allison and Martiny, 2008). And although we identified changes in taxa for example associated with nutrient cycling to be enriched under BR fertilizers, we can only speculate about their actual role in our experiment. Thus, to complement our findings we further analyzed plant yield and basal respiration as a measure for soil activity.

In our study the grass-clover mixture growing on soil amended with BR fertilizers produced the highest plant yield (Figure 4B), although only soil treatment with BR+ was statistically significant compared to the no-fertilizer treatment. Not only abiotic but also biotic components of BSFL residues might have been the drivers, but soils treated with BR+ and BR-did not differ significantly in yield, which would be necessary to separate the effects. The availability of plant nutrients had probably only a minor effect on yield, since correlations between DWY and NO_3_^−^ concentrations in soil were weak. Differences in P and K amounts added with the fertilizers were probably negligible considering the high nutritional status of the soil. This corresponds to previous studies suggesting an improvement of plant performance by BSFL residues that is not mediated by a direct supply of plant available nutrients. Chirere et al. (2021) and Klammsteiner et al. (2020a) could not find significant differences in yield of Swiss Chard and Perennial Ryegrass, respectively, when comparing BSFL residues to mineral fertilizer, however the latter significantly increased the mineral N content of the soil. Chirere et al. (2021) suggested the stimulatory capacity of humic acids in BSFL residues to contribute to plant yield development but they did not quantify their abundance. Also derivates of chitin were suggested to stimulate plant growth directly (Winkler et al., 2017; Xu and Mou, 2018) and are probably present in BSFL residues as discussed before. Screening and quantification of potential plant growth promoting substances across BSFL residues of distinct origin is certainly needed to advance the discussion. However, not only abiotic components of BSFL residues, but also microbes enriched in soil *via* BSFL residues could have had a positive influence on soil fertility. Similarly to the beforementioned, Gebremikael et al. (2022) and Tan et al. (2021) reported that fertilization with BSFL residues led to a better plant performance than expected from their supply with plant-available nutrients, whereas they suggest an increased stimulation by plant growth promoting microbes under BSFL residue treatment. Poveda et al. (2019) argued that plants can benefit from mealworm residue application as a fertilizer by the introduction of a variety of plant growth promoting rhizobacteria. The fungal taxon *Mortierella elongata* that was thriving in our soils fertilized with BR+ and BR- (Supplementary Table 6) is associated with plant growth promotion *via* the stimulation of phytohormone production (Li et al., 2018). Members of the bacterial genus *Bacillus*, a bacterial group that harbors many plant growth promoting strains (Saxena et al., 2020), was highly enriched under soil amended with BR+ and could have played a direct role in the significant yield increase under BR+ treatment. However, it is important to note that a comprehensive number of metabolically diverse species belongs to this genus. Again, the soil used in our experiment had a high nutritional status and we expect microbes associated with BSFL residues to promote crop yield more clearly under resource poor conditions, where rhizosphere microbiomes tend to show an increasing impact on plant performance (Van Der Heijden et al., 2008).

Microbial heterotrophic activity is another potential indicator of soil fertility and can be estimated by soil respiration (Robertson et al., 1997). An increased basal respiration might arise from both an increase in microbial activity and microbial biomass, which we, however, did not disentangle here. Gebremikael et al. (2022) showed that application of BSFL residues produced on different organic wastes can increase soil microbial biomass more strongly than an anaerobic digestate of food waste, which suggests that an increase in soil microbial biomass was likely contributing to basal respiration in our study. We could show that non-sterile BSFL residues can stimulate the soil microbiome stronger than a conventional compost. The application of BR+ increased basal respiration compared to the application of CC fertilizers (Figure 4A). This is in line with Watson et al. (2021), who observed even stronger differences in cumulated soil respiration between the addition of BSFL residues and vermicompost in a 28-day soil incubation experiment. However, their fertilization rates were around 10 times higher compared to our study, and we measured microbial activity later after fertilizer application (45 days). Watson et al. (2021), suggested that the main driver of differences in basal respiration among treated soils was labile C, a key fuel for microbial activity, and probably depleted in CC compared to BR fertilizers. However, in line with beforementioned assumptions we would propose that original labile C added to soil through BR fertilizers was likely to be majorly consumed after 45 days and thus had probably a negligible effect on basal respiration. Thus, we would assume that rather the C sources released from the slower, fungi-dominated decomposition of cellulose or lignin (Insam and De Bertoldi, 2007) probably inherent to BSFL residues were driving basal respiration in our experiment. In fact, fertilization with BR fertilizers led to an enrichment of members of fungal taxa that were previously associated with the decomposition of organic matter such as genus *Mortierella* (Jayasinghe and Parkinson, 2008) and family *Lasiosphaeriaceae* (Nordén and Paltto, 2001). That might have led to a trickle-down effect with their metabolic products serving as a substrate for other microbial taxa resulting in an increased basal respiration. Again, it is not possible to clearly conclude that microbes introduced with BR+ contributed to the stimulation of soil microbes since BR- and BR+ were not differing significantly in basal respiration.

In this study we combined the sterilization of organic soil inputs with physicochemical and microbial assessments. This method can serve as an example to test the validity of other soil amendments to which a positive influence of inherent microorganisms on soil fertility is attributed, also referred to as “biofertilizers” (Mahanty et al., 2017).

## Conclusion

Fertilization with BSFL residues altered the soil microbial community and tended to increase microbial activity and crop yield when compared to compost-amended or unamended soils, respectively. By including a non-thermal soil sterilization treatment, we could provide indication that these effects were partially mediated by the introduction of BSFL residue-derived microbes to the soil. Among these, taxa commonly associated with organic matter decomposition and plant growth promotion were identified, which could also have potential downstream effects on other bacterial and archaeal taxa important for soil fertility. In contrast, the BSFL residues had limited influence on the measured soil physicochemical properties, although we assume that organic compounds characteristic for BSFL residues used in our experiment such as chitin and its derivates with promising applications in agricultural production might have been enriched upon amendment. Thus, we can assume that both biotic effects *via* the introduced organisms as well as abiotic effects *via* bioactive substances can influence soil fertility and plant performance. Therefore, BSFL residues with high plant-nutritional value and microbial capabilities to promote plant growth have the potential to improve sustainable agricultural production, especially in low-income countries, where BSFL rearing is meeting multiple needs as a promising, low-threshold technology in circular economy. Notably the characteristics of relevant biotic and abiotic components in BSFL residues are likely to be impacted by their post-processing such as composting and should further be assessed to inform the debate with high practical significance. To corroborate ours and other findings, further research should focus on the quantification of chitin, labile carbon, fiber and compounds like humic acids in BSFL residues. Results on changes in soil microbial community structure should be complemented with analyses on the functional capacity of the microbial community, for example by measuring the genetic potential *via* quantification of key genes through quantitative PCR, metagenome sequencing to identify genes that are enriched under BSFL residue amendment, and extracellular enzyme assays to directly measure shifts in specific enzymatic activities. The results of our study are specific to our experimental setup since the composition of BSFL residues both in terms of biota and substrate composition differ largely according to their origin and processing. Soil type, crop type, and investigated time scale are other important factors that need to be considered further, as these parameters were fixed in our experiment. Studies on long-term effects of such amendments, especially under field conditions, are also largely missing. All these investigations can pave the way for using organic fertilizers derived from insect rearing on waste products for a circular and sustainable agriculture.

## Data availability statement

The datasets presented in this study can be found in online repositories. The names of the repository/repositories and accession number(s) can be found at: https://www.ebi.ac.uk/ena, PRJEB54639.

## Author contributions

AF, BW, MG, AM, and MH have conceived the study. AF conducted the greenhouse experiment. AF and RF performed the laboratory analyses. BW, SK, MK, BM and LS organized sourcing and shipping of fertilizers. AF, BW and MH analysed the data. AF wrote the initial draft of the manuscript. BW, MG and MH provided substantial input. All authors contributed to the article and approved the submitted version.

## Funding

The RUNRES project funded by the Swiss Agency for Development and Cooperation (project number 7F09521) provided us with the applied organic fertilizers through the two participating Rwandan bioconversion companies COPED and Maggot Farm Production Ltd. Open access funding provided by ETH Zurich.

## Conflict of interest

BM is employed by Maggot Farm.

The remaining authors declare that the research was conducted in the absence of any commercial or financial relationships that could be construed as a potential conflict of interest.

## Publisher’s note

All claims expressed in this article are solely those of the authors and do not necessarily represent those of their affiliated organizations, or those of the publisher, the editors and the reviewers. Any product that may be evaluated in this article, or claim that may be made by its manufacturer, is not guaranteed or endorsed by the publisher.

## Footnotes

^1^ https://www.runres.ethz.ch
