# Supplementary material for: Residues from black soldier fly (Hermetia illucens) larvae rearing influence the plant-associated soil microbiome in the short term
Source: Front Microbiol. 2022 Sep 26;13:994091. doi: 10.3389/fmicb.2022.994091 (PMC9550165; doi:10.3389/fmicb.2022.994091)
Supplement: Supplementary file 1 [file Data_Sheet_1.pdf]

Supplementary Material

1 Supplementary Figures and Tables

1.1 Supplementary Tables

Supplementary Table 1. Effects of fertilizer application on the physicochemical soil characteristics at each sampling timepoint as determined by ANOVA or Friedmann-test, respectively. Significant values (p<0.05) are indicated in bold.

Parameter [1]	Day 0		Day 24		Day 42	
	F / $\chi 2$ [2]	P	F / $\chi 2$	P	F / $\chi 2$	P
NO_3^- -N	12.8	**< 0.001**	15.9	**< 0.001**	6.1$_{SB}$	**0.004$_{SB}$**
NH_4^+-N	14.7$_{NN}$	**0.005$_{NN}$**	7.5$_{NN}$	0.111$_{NN}$	10.7$_{NN}$	**0.029$_{NN}$**
pH	0.5	0.752	11.4$_{NN}$	**0.023$_{NN}$**	7.7$_{NN}$	0.105$_{NN}$
EC	17.8	**0.001**	17.1$_{NN}$	**0.002$_{NN}$**	3.3	**0.038**
N	0.7	0.594	5.4	**0.006**	6.6$_{NN}$	0.161$_{NN}$
C	1.7	0.209	2.1	0.131	0.7	0.619
C:N	3.0$_{NN}$	0.551$_{NN}$	2.6	0.072	5.1$_{NN}$	0.275$_{NN}$
GWC	1.7	0.196	0.2	0.938	1.6$_{SB}$	0.231$_{SB}$
DMY					3.1$_{SB}$	**0.045$_{SB}$**
Basal Resp.					5.9$_{SB}$	**0.004$_{SB}$**
CEC_{eff}					1.3	0.305

[1] Nitrate (NO_3^--N), ammonium (NH_4^+-N), pH (in H_2O), electrical conductivity (EC), total nitrogen (N), total carbon (C), C : N (C:N), gravimetric water content (GWC), dry weight yield (DWY), basal respiration (Basal resp.), effective cation exchange capacity (CEC_{eff}). The last three parameters were only measure at harvest.

[2] F-ratios from ANOVA and χ^2 from Friedmann-test where applicable, respectively. $_{NN}$ indicates non-normal distribution of residuals and thus application of Friedmann-test. Factors included the fertilizer application (degrees of freedom = 4) and block (degrees of freedom = 4). $_{SB}$ indicates significant block effect.

Supplementary Table 2. Physicochemical soil characteristics by sampling date and fertilizer application. Values are based on dry weights (average ± standard deviations, n = 5).

Day	Treatment	Parameter[1,2]										
		NO_3^--N [mg kg^{-1}]	NH_4^+-N [mg kg^{-1}]	EC [µS cm^{-1}]	pH	N [g kg^{-1}]	C [g kg^{-1}]	C:N [g g^{-1}]	GWC [g g^{-1}]	CEC_{eff} [$cmol_c$ kg^{-1}]	DWY [g]	Basal Resp. [µmol g^{-1} h^{-1}]
0	sBR+	69.4 ± 4.3 bc[2]	1.2 ± 0.1 AB	697 ± 29 b	7.0 ± 0.0	2.9 ± 0.2	45.1 ± 2.0	15.9 ± 1.5	0.26 ± 0.01			
0	sBR-	79.1 ± 5.3 c	1.5 ± 0.1 A	723 ± 11 b	7.1 ± 0.1	3.1 ± 0.4	44.6 ± 2.2	14.4 ± 2.2	0.25 ± 0.01			
0	sCC+	53.9 ± 7.8 ab	1.0 ± 0.1 BC	646 ± 6 a	7.0 ± 0.0	2.8 ± 0.7	46.9 ± 3.1	18.1 ± 7.0	0.27 ± 0.00			
0	sCC-	67.6 ± 11.3 bc	0.9 ± 0.1 C	661 ± 6 a	7.1 ± 0.0	2.8 ± 0.2	42.9 ± 2.9	15.5 ± 0.8	0.26 ± 0.01			
0	sN0	47.0 ± 6.5 a	2.7 ± 3.2 A	631 ± 7 a	7.1 ± 0.0	2.8 ± 0.2	43.1 ± 3.7	15.6 ± 1.0	0.25 ± 0.02			
24	sBR+	85.8 ± 7.2 b	0.7 ± 0.1	733 ± 5 A	7.0 ± 0.0 ABC	3.3 ± 0.5 ab	45.9 ± 2.9	14.3 ± 2.5	0.33 ± 0.02			
24	sBR-	91.4 ± 7.4 b	0.9 ± 0.1	738 ± 27 A	7.0 ± 0.0 AB	3.0 ± 0.1 a	45.4 ± 2.1	15.0 ± 0.7	0.34 ± 0.02			
24	sCC+	63.2 ± 10.7 a	1 ± 0.4	657 ± 10 B	7.1 ± 0.0 A	3.0 ± 0.1 a	43.1 ± 2.2	14.4 ± 1.1	0.33 ± 0.02			
24	sCC-	67.3 ± 8.2 a	0.8 ± 0.2	664 ± 53 BC	7.0 ± 0.0 BC	2.9 ± 0.2 a	42.9 ± 2.5	14.8 ± 1.4	0.34 ± 0.03			
24	sN0	58.1 ± 5.8 a	0.9 ± 0.1	691 ± 17 C	7.0 ± 0.0C	3.7 ± 0.5 b	44.6 ± 2.5	12.1 ± 2.0	0.33 ± 0.03			
42	sBR+	35.6 ± 14.4 bc	1.1 ± 0.0 AB	573 ± 32	7.0 ± 0.0	2.9 ± 0.1	44.0 ± 0.7	15.0 ± 0.6	0.26 ± 0.03	24.4 ± 0.82	6.3 ± 0.5 b	0.059 ± 0.007 b
42	sBR-	37.5 ± 16.6 c	1.1 ± 0.1 AB	585 ± 34	7.0 ± 0.0	2.6 ± 0.7	44.3 ± 1.0	18.3 ± 7.06	0.26 ± 0.04	23.99 ± 0.85	6.0 ± 1.1 ab	0.053 ± 0.01 ab
42	sCC+	18.9 ± 7.8 ab	1.0 ± 0.2 A	540 ± 24	7.1 ± 0.0	2.9 ± 0.2	44.5 ± 1.7	15.6 ± 1.3	0.25 ± 0.06	22.86 ± 2.38	5.9 ± 0.9 ab	0.048 ± 0.007 a
42	sCC-	23.5 ± 6.3 ac	1.0 ± 0.0 A	538 ± 25	7.1 ± 0.0	3.0 ± 0.3	43.8 ± 1.9	15.0 ± 1.9	0.26 ± 0.04	23.29 ± 0.97	5.7 ± 0.6 ab	0.044 ± 0.007 a
42	sN0	15.8 ± 5.2 a	1.2 ± 0.1 B	555 ± 11	7.0 ± 0.0	3.1 ± 0.0	43.1 ± 0.8	14.0 ± 0.3	0.29 ± 0.02	23.89 ± 0.61	5.4 ± 0.5 a	0.051 ± 0.004 ab

[1] Nitrate-N (NO_3^- -N), ammonium-N (NH_4^+-N), electrical conductivity (EC), pH (pH in H_2O), total nitrogen (N), total carbon (C), C:N, gravimetric water content (GWC), CEC_{eff} (effective cation exchange capacity), dry weight yield of shoots not corrected for significant block effect (DWY) and basal respiration not corrected for significant block effect (Basal Resp.).

[2] Distinct lower-case letters indicate differences between treatments at the same sampling day($p < 0.05$) according to Tukey´s HSD test. Distinct capital-case letters indicate differences between treatments at the same sampling day ($p < 0.05$) according to Conover post-hoc test.

Supplementary Table 3. Effects of fertilizer treatment on microbial alpha-diversity determined by PERMANOVA Significant values (p<0.05) are indicated in bold.

Test[1]	Bacteria & Archaea			Fungi		
	Observed Richness	Shannon Diversity	Pielou's Evenness	Observed Richness	Shannon Diversity	Pielou's Evenness
	F (P)[2]	F (P)	F (P)	F (P)	F (P)	F (P)
Treatment	15.6 **(0.001)**	11.6 (**0.001)**	7.7 (**0.001)**	5.9 **(0.001)**	1.5 (0.244)	1.1 (0.390)
Sampling day	1.1 (0.321)	6.5 (**0.008)SD**	10.2 (**0.001)SD**	2.2 (0.143)	3.3 (0.059)	3.3 (**0.040**)
Treatment x Sampling day	1.5 (0.196)	2.0 (0.078)	2.2 (0.054)	1.5 (0.203)	1.1 (0.424)	1.1 (0.393)

[1] Effects of factors and their interaction were analyzed by univariate permutational analysis of variance (PERMANOVA). Factors are fertilizer treatment (degrees of freedom = 4, sBR+, soil fertilized with BSFL residue; sBR-, soil fertilized with sterile BSFL residue; sCC+, soil fertilized with conventional compost; sCC-, soil fertilized with sterile conventional compost; sN0, no-fertilizer treatment soil), day of sampling (degrees of freedom = 2, day 0, 24, 42) whereas their interaction has 8 degrees of freedom. Values represent the pseudo-F ratio (F) and the level of significance (P). Values at P <0.05 are shown in bold. SD indicates significant dispersion.

Supplementary Table 4. Alpha diversity metrics by soil characteristics by sampling date and fertilizer application (average ± standard deviations, n = 5).

Sampling day	Treatment[1]	Bacteria & Archaea			Fungi		
		Observed Richness	Shannon Diversity	Pielou's Evenness	Observed Richness	Shannon Diversity	Pielou's Evenness
0	sBR+	8725 ± 101[2]	8.06 ± 0.02	0.888 ± 0.001	927 ± 71	4.74 ± 0.41	0.694 ± 0.053
0	sBR-	8630 ± 141	8.05 ± 0.04	0.888 ± 0.003	934 ± 66	4.68 ± 0.19	0.685 ± 0.024
0	sCC+	8476 ± 134	8.02 ± 0.03	0.887 ± 0.002	947 ± 40	4.83 ± 0.47	0.705 ± 0.066
0	sCC-	8531 ± 80	8.02 ± 0.02	0.887 ± 0.002	982 ± 52	4.99 ± 0.18	0.725 ± 0.021
0	sN0	8346 ± 83	7.97 ± 0.02	0.883 ± 0.001	976 ± 27	5.01 ± 0.02	0.729 ± 0.005
24	sBR+	8596 ± 77	8.03 ± 0.02	0.886 ± 0.002	953 ± 59	4.92 ± 0.27	0.717 ± 0.034
24	sBR-	8495 ± 160	8.03 ± 0.05	0.888 ± 0.004	902 ± 36	4.85 ± 0.03	0.713 ± 0.005
24	sCC+	8474 ± 158	8 ± 0.04	0.885 ± 0.003	971 ± 31	4.91 ± 0.2	0.714 ± 0.026
24	sCC-	8603 ± 42	8.05 ± 0.01	0.889 ± 0.001	978 ± 78	4.82 ± 0.5	0.699 ± 0.065
24	sN0	8403 ± 96	7.99 ± 0.02	0.885 ± 0.001	916 ± 22	5.01 ± 0.11	0.735 ± 0.014
42	BR+	8731 ± 84	8.07 ± 0.02	0.89 ± 0.001	990 ± 14	5.08 ± 0.03	0.737 ± 0.005
42	BR-	8528 ± 67	8.06 ± 0.01	0.89 ± 0.001	905 ± 48	4.86 ± 0.19	0.714 ± 0.022
42	CC+	8522 ± 72	8.03 ± 0.02	0.888 ± 0.002	1010 ± 21	5.16 ± 0.04	0.746 ± 0.007
42	sCC-	8568 ± 104	8.04 ± 0.01	0.888 ± 0.001	1005 ± 37	5.09 ± 0.09	0.736 ± 0.011
42	sN0	8440 ± 67	8.03 ± 0.01	0.888 ± 0.001	944 ± 32	4.93 ± 0.15	0.719 ± 0.019

[1] sBR+, soil fertilized with BSFL residue; sBR-, soil fertilized with sterile BSFL residue; sCC+, soil fertilized with conventional compost; sCC-, soil fertilized with sterile conventional compost; sN0, no-fertilizer treatment

[2] No significant differences between sampling day x treatment combinations were found by pairwise comparisons (PERMANOVA) correcting for multiple comparisons using the Benjamini-Hochberg method.

Supplementary Table 5. Taxonomic assignment and read counts (average ± standard deviations, n = 5) of sensitive bacterial ASVs.

LCA[1]	sBR+[3]	sBR-	sCC+	sCC-	sN0	F[2]	Q	P
f:Bacillaceae°*	22.8 ± 6.4[4]	8.2 ± 2.6	1 ± 1	0.6 ± 0.9	0.6 ± 0.9	45.59	0.04	0.001
g:Bacillus°*	28.9 ± 7.8	5.7 ± 1.3	4.8 ± 8.7	0.8 ± 1.1	0.4 ± 0.5	25.18	0.04	0.001
g:Actinopolymorpha°*	0 ± 0	0.2 ± 0.4	40.6 ± 21.8	20.8 ± 11.9	0 ± 0	13.4	0.04	0.001
f:67-14	74.3 ± 10.4	108.8 ± 4.1	87.2 ± 10.3	86.1 ± 3.1	102.8 ± 13.5	11.41	0.04	0.001
f:Chitinophagaceae	104.6 ± 8.1	46.6 ± 5.3	74.4 ± 19.1	82 ± 24.2	57 ± 9.3	11.25	0.04	0.001
f:Nitrososphaeraceae	155.7 ± 11.5	96.7 ± 13.3	129.7 ± 12.7	122.8 ± 24.9	113.6 ± 7.1	10.37	0.04	0.001
f:Intrasporangiaceae	6.8 ± 1.1	10 ± 2.3	10.2 ± 2.3	8.2 ± 2.2	13.8 ± 1.3	9.48	0.04	0.001
f:Chitinophagaceae	46.1 ± 7.6	20.9 ± 3.7	29.6 ± 7.7	28.6 ± 9.9	22.4 ± 6.2	9.33	0.04	0.001
*g:Bacillus**	44.6 ± 4.7	26.4 ± 9.8	43.8 ± 8	33.8 ± 6.3	24.6 ± 4.7	8.98	0.04	0.001
g:Dinghuibacter	13.4 ± 3.8	8.2 ± 3	17.8 ± 3.1	14.9 ± 4.2	7 ± 3.1	8.55	0.04	0.001
f:Pirellulaceae	17.4 ± 4.3	7.8 ± 2.2	11.4 ± 2.1	10.8 ± 2	12.8 ± 2.4	8.16	0.04	0.001
g:Dinghuibacter	17 ± 3.4	8.8 ± 0.8	12.4 ± 3	12.9 ± 1.1	9.5 ± 3.5	7.59	0.04	0.001
g:Iamia	19.2 ± 3.1	23.4 ± 3.8	17.1 ± 3.6	20.2 ± 2.6	27 ± 2.8	7.23	0.04	0.001
*o:RBG-13-54-9**	28.2 ± 4.3	13.8 ± 2.4	20.1 ± 4.4	22.2 ± 6.4	20.2 ± 3.2	6.96	0.04	0.001
f:Blastocatellaceae	35.6 ± 6.3	18.8 ± 3.9	22.4 ± 4.9	28.5 ± 7.3	20.9 ± 5.9	6.9	0.04	0.001
g:Ohtaekwangia	9.8 ± 2.2	5.2 ± 2.7	7.4 ± 1.9	5 ± 1.4	4.2 ± 1.5	6.52	0.04	0.001

[1] LCA indicating the lowest taxonomic rank and the corresponding taxon name that was assigned to the respective ASV. Preposed letters indicate taxonomic rank. f:, family; g: genus. * or ° indicates whether ASV was identified in the fertilizers BR, BSFL residues or CC, conventional compost themselves.

[2] Pseudo F-ratios from PERMANOVA, p-values and q-values controlling for type I error inflation due to multiple testing. Only ASVs with q < 0.05 from PERMANOVA and q > 0.05 for dispersion are shown in this table.

[3] sBR+, soil fertilized with BSFL residue; sBR-, soil fertilized with sterile BSFL residue; sCC+, soil fertilized with conventional compost; sCC-, soil fertilized with sterile conventional compost; sN0, non-fertilized soil

[4] Read counts are based on an iterative (100 iterations) subsampling approach of the ASV matrices to remove systematic biases in read counts potentially arising from the sequencing workflow.

Supplementary Table 6. Taxonomic assignment and read counts (average ± standard deviations, n = 5) of sensitive fungal ASVs.

LCA[1]	sBR+[3]	sBR-	sCC+	sCC-	sN0	F[2]	Q	P
*g:Staphylotrichum°**	67.8 ± 14.5[4]	215.2 ± 37.3	80.6 ± 23.5	59.4 ± 14.9	59.8 ± 7.2	46	0.01	0.001
p:Ascomycota	22.6 ± 11.9	73.8 ± 24.3	2.6 ± 1.8	3.2 ± 2.7	2.8 ± 1.1	31.6	0.01	0.001
f:Lasiosphaeriaceae	57.5 ± 24.5	89.9 ± 30.8	9.5 ± 10.4	4 ± 5.7	2.4 ± 2.1	22.8	0.01	0.001
*g:Mortierella°**	866.2 ± 115	838.5 ± 122.2	544.4 ± 48.5	571.5 ± 100.5	428.6 ± 66.4	20.6	0.01	0.001
g:Mortierella	45.7 ± 18	103.4 ± 33.6	32.8 ± 17.9	35.3 ± 10.1	16.6 ± 6.3	14.5	0.01	0.001
*g:Staphylotrichum°**	374.4 ± 40.2	838.3 ± 182.3	485.3 ± 88	514.5 ± 131.2	456.8 ± 69.9	12.2	0.01	0.001
*s:Acremonium persicinum°**	111.8 ± 16.4	100.4 ± 12.2	147.4 ± 25.9	184.2 ± 17.5	152.6 ± 30.5	12.2	0.01	0.001
*g:Lecythophora°**	250.4 ± 46.9	236.4 ± 40.6	363.6 ± 61.2	409.2 ± 54.3	391.6 ± 55.2	12.1	0.01	0.001
*g:Botryotrichum**	103.9 ± 49.4	60.3 ± 47.6	0.7 ± 1.1	2.2 ± 2.5	1.8 ± 2.4	11.6	0.01	0.001
p:Basidiomycota	41.8 ± 8.3	29.4 ± 7.1	67.2 ± 12.9	54.1 ± 6	65.7 ± 17	10.6	0.01	0.001
g:Mortierella°	161.5 ± 53.3	143.7 ± 21.5	84.8 ± 32.3	67.2 ± 29.2	54.3 ± 13.5	10.5	0.01	0.001
*o:Pleosporales°**	196.5 ± 14	130.5 ± 17.6	292.4 ± 57.6	331.1 ± 92.2	264 ± 60.5	10	0.01	0.001
g:Trichoderma	34.2 ± 2.2	33.4 ± 5	62.7 ± 14.9	45.7 ± 10.1	61 ± 12.1	9.9	0.01	0.001
o:Sordariales	83.8 ± 64.9	185 ± 51.6	55.6 ± 11.3	74.1 ± 29.5	52.4 ± 2.1	9.5	0.02	0.002
p:Ascomycota	20.2 ± 7.7	21.7 ± 8.3	52.2 ± 11.6	38 ± 5.3	29.7 ± 13.3	9.2	0.01	0.001
*f:Trichosporonaceae°**	331.6 ± 20.8	264.8 ± 56.7	540.1 ± 147	431.5 ± 49.9	381.7 ± 49.4	9	0.01	0.001
s:Mucor exponens°	100.7 ± 19.8	53.2 ± 10.4	127 ± 38.6	155.3 ± 42.9	90.1 ± 21.1	8.7	0.03	0.003
g:Mortierella	37.4 ± 9.9	62.6 ± 31.5	17.5 ± 9.1	15.5 ± 7.9	13.6 ± 6.6	8.6	0.01	0.001
s:Mucor circinelloides°*	20 ± 10.8	2 ± 2.3	2.4 ± 1.5	8.6 ± 8.3	1.8 ± 2.2	7.8	0.02	0.002
p:Ascomycota°	51.7 ± 9.5	79.5 ± 29.6	97.4 ± 11.9	111.8 ± 23.8	110.1 ± 21.1	7.4	0.01	0.001
f:Lasiosphaeriaceae	2 ± 1.2	1 ± 0.7	5.2 ± 2.3	6.6 ± 3.2	3.4 ± 0.9	7.1	0.03	0.003
o:Pleosporales	44.4 ± 7.6	29 ± 7.8	53 ± 7.3	56.4 ± 14.8	40.8 ± 5	7	0.01	0.001
s:Leucosporidium intermedium	3 ± 1.4	3.8 ± 2.5	13.9 ± 5.8	5.9 ± 2.8	5.8 ± 4.2	7	0.02	0.002
f:Cordycipitaceae°*	74 ± 11.3	72 ± 14	101.6 ± 17.8	108 ± 9.4	90 ± 14.7	6.8	0.03	0.004
f:Schizoporaceae°*	260.4 ± 31.2	178.7 ± 26.7	301.2 ± 45.5	309.5 ± 48.9	264.1 ± 61.8	6.7	0.02	0.002
f:Halosphaeriaceae	57.6 ± 10.5	23.4 ± 16.1	74 ± 24.5	57.2 ± 13.9	47 ± 11.6	6.7	0.03	0.003
o:Chaetothyriales	1.7 ± 1	1.8 ± 1.5	2.3 ± 1.9	2.2 ± 1.1	8.2 ± 4.6	6.6	0.01	0.001
g:Podospora	9.8 ± 6.6	72.9 ± 59.2	4 ± 5.1	1.6 ± 1.5	2.2 ± 1.6	6.6	0.01	0.001
s:Mortierella elongata	13.4 ± 5.5	16.4 ± 7.2	8.6 ± 4.1	5.8 ± 3.5	3.6 ± 1.8	6.2	0.04	0.005
g:Cylindrocarpon	9.2 ± 2.8	9.7 ± 4.3	20.2 ± 4.7	15.6 ± 3.8	15.8 ± 5.1	6.1	0.03	0.003
p:Ascomycota	74.2 ± 14.6	51.2 ± 11.3	99.6 ± 18.6	97.9 ± 32.6	64 ± 12.4	5.9	0.03	0.003
*o:Hypocreales**	44.3 ± 6.8	34.5 ± 5.6	49.5 ± 11	55.8 ± 2.6	49.8 ± 9.4	5.5	0.03	0.003
o:Lobulomycetales	12.4 ± 5	6.6 ± 6.1	21.2 ± 8.5	18.9 ± 5	9.8 ± 3.8	5.4	0.04	0.005
o:Hypocreales	38.6 ± 4.6	35.1 ± 14	34.4 ± 10.3	59 ± 10.4	67.4 ± 25.6	5.4	0.02	0.002
s:Mucor moelleri	4.4 ± 1.1	5 ± 1.4	9.4 ± 3.6	5.9 ± 3	2.8 ± 1.9	5.1	0.03	0.004
f:Lasiosphaeriaceae	9.7 ± 9.5	107 ± 124.9	0.6 ± 1.3	0.8 ± 1.8	0.6 ± 0.9	3.5	0.01	0.001
p:Ascomycota	13.6 ± 5.2	223.3 ± 269.3	7.4 ± 4.6	10.6 ± 10.8	7.6 ± 7.8	3.1	0.02	0.002
s:Stachybotrys sansevieriae	6.8 ± 1.6	7.6 ± 2.3	9.4 ± 4	14.8 ± 6.3	25.4 ± 21.1	2.9	0.02	0.002
d:Fungi	120.9 ± 131.7	36.4 ± 18.2	17.4 ± 6.6	24.1 ± 8.4	35.2 ± 25	2.4	0.03	0.003
f:Lasiosphaeriaceae	11.8 ± 6.9	46.6 ± 61.5	5.4 ± 6.3	3.6 ± 2.5	2.2 ± 2.3	2.2	0.05	0.006
*s:Phaeosphaeria caricis°**	0 ± 0	0 ± 0	18.2 ± 29.7	0 ± 0	0 ± 0	1.9	0.01	0.001
s:Cephaliophora tropica°*	24.4 ± 43	0 ± 0	0 ± 0	0.2 ± 0.4	0 ± 0	1.6	0.02	0.002

[1] LCA indicating the lowest taxonomic rank and the corresponding taxon name that was assigned to the respective ASV. Preposed letters indicate taxonomic rank. d:,domain; p:,phylum; o:,order; f:, family; g: genus; s:,species * or ° indicates whether ASV was identified in the fertilizers BR, BSFL residues or CC, conventional compost themselves.

[2] Pseudo F-ratios from PERMANOVA, p-values and q-values controlling for type I error inflation due to multiple testing. Only ASVs with q < 0.05 from PERMANOVA and q > 0.05 for dispersion are shown in this table.

[3] sBR+, soil fertilized with BSFL residue; sBR-, soil fertilized with sterile BSFL residue; sCC+, soil fertilized with conventional compost; sCC-, soil fertilized with sterile conventional compost; sN0, non-fertilized soil

[4] Read counts are based on an iterative (100 iterations) subsampling approach of the ASV matrices to remove systematic biases in read counts potentially arising from the sequencing workflow.

1.2 Supplementary Figures

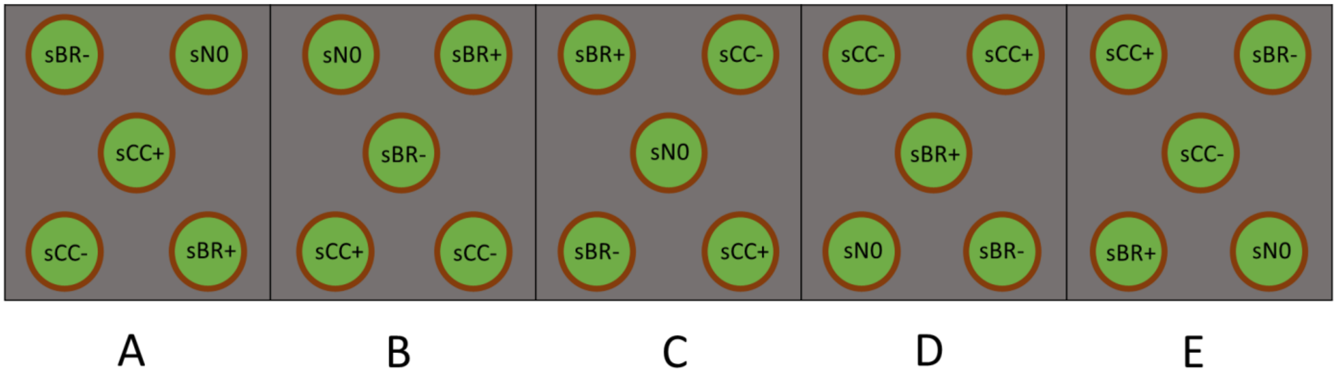

Supplementary Figure 1. Experimental design of the greenhouse pot trial. A, B, C, D, and E indicate the location of blocks. Circles represent the pots and are labelled with the respective treatment.

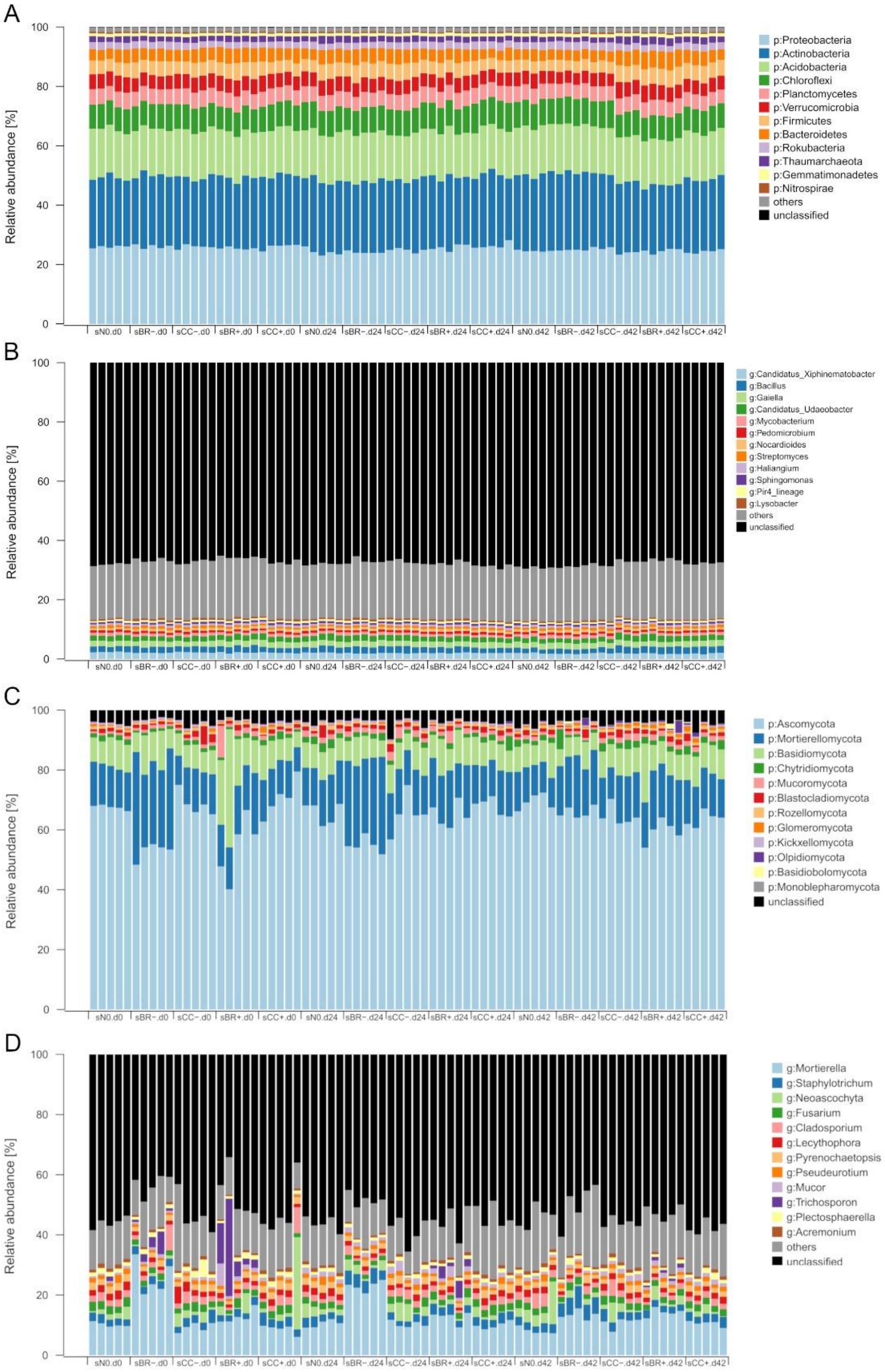

Supplementary Figure 2. Relative abundances of most frequently observed ASVs by assigned taxa for each pot at each sampling day (treatment x sampling day combinations indicated at x-axis). For bacteria & archaea (phylum: A, genus: B) and fungi (phylum: C ,genus D). Sampling days 0, 24 and 42 and treatments (sBR+, soil fertilized with BSFL residue; sBR-, soil fertilized with sterile BSFL residue; sCC+, soil fertilized with conventional compost; sCC-, soil fertilized with sterile conventional compost; sN0, non-fertilized soil). The variance explained by each PCO axis is given in parentheses.

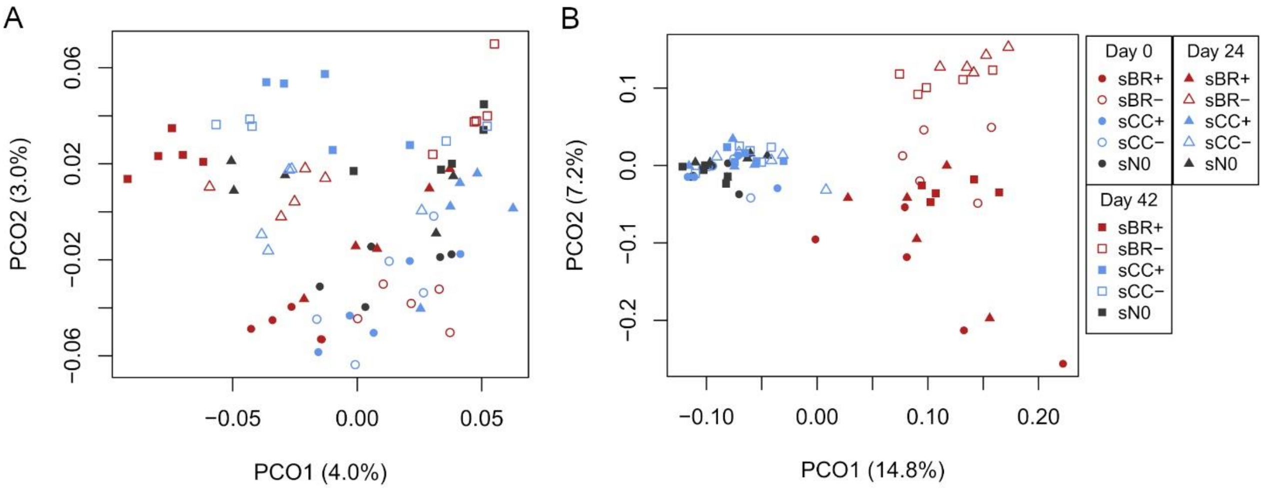

Supplementary Figure 3. Fertilization effects on bacterial/archaeal (A) and fungal (B) soil community structures. First and second axis of PCoA (principal coordinate analysis) show principal variance components based on Bray-Curtis dissimilarity at sampling days 0 (circles), 24 (triangles) and 42 (squares) and treatments (sBR+, soil fertilized with BSFL residue; sBR-, soil fertilized with sterile BSFL residue; sCC+, soil fertilized with conventional compost; sCC-, soil fertilized with sterile conventional compost; sN0, non-fertilized soil). The variance explained by each PCO axis is given in parentheses. for bacteria and archaea (A) and fungi (B).

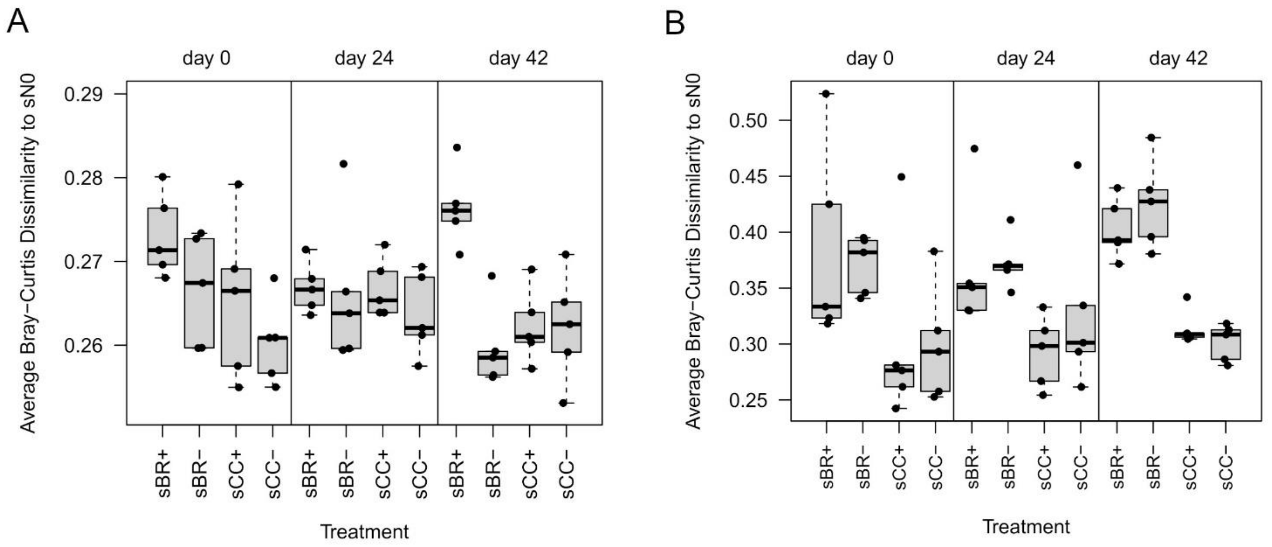

Supplementary Figure 4. Dissimilarity between the average soil bacterial (A) and fungal (B) composition of the non-fertilized soil (sN0) and fertilized soil at the same sampling day. sBR+, soil fertilized with BSFL residue; sBR-, soil fertilized with sterile BSFL residue; sCC+, soil fertilized with conventional compost; sCC-, soil fertilized with sterile conventional compost. Sampling of soil was conducted at days 0, 24 and 42 of the experiment.

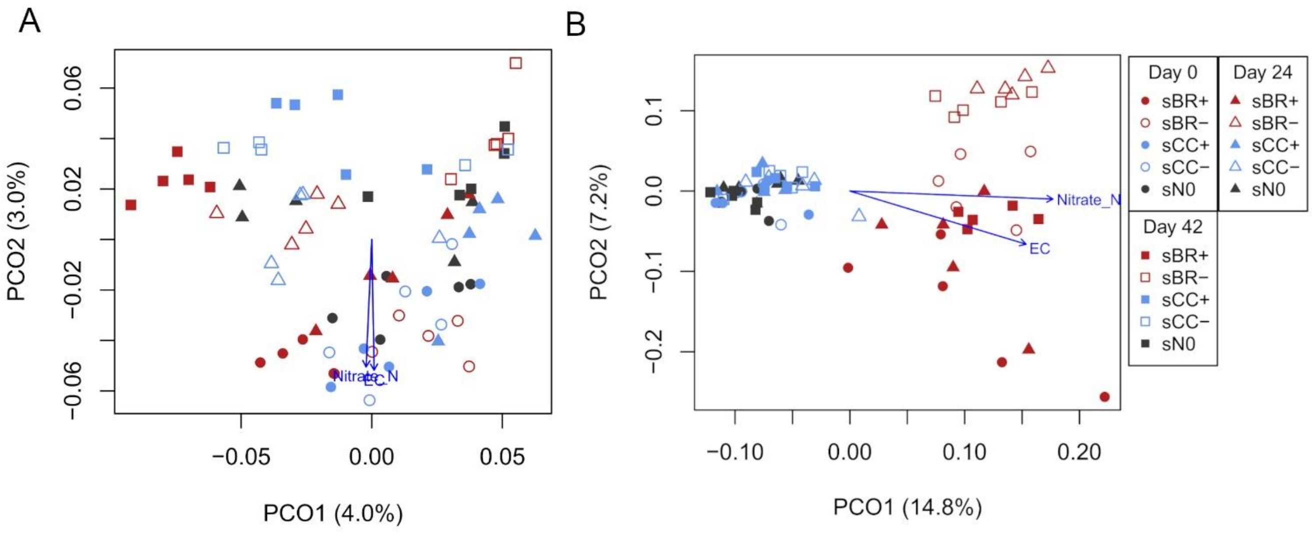

Supplementary Figure 5. Fertilization effects on soil bacterial/archaeal (A) and fungal (B) community structures and physicochemical properties (nitrate-N content, ammonium-N content, electrical conductivity, pH in H_2O, total nitrogen, total carbon, total nitrogen:total carbon ratio). First and second axis of PCO (principal coordinate analysis) show principal variance components based on Bray-Curtis dissimilarity at sampling days 0 (circles), 24 (triangles) and 42 (squares) and treatments (sBR+, soil fertilized with BSFL residues; sBR-, soil fertilized with sterile BSFL residues; sCC+, soil fertilized with conventional compost; sCC-, soil fertilized with sterile conventional compost; sN0, non-fertilized soil). The variance explained by each PCO axis is given in parentheses for bacteria and archaea (A) and fungi (B). Significantly correlated ($p<0.05$) physicochemical variables (EC, Electrical conductivity and Nitrate_N, Nitrate-N content) were fitted onto ordinations.
